# Multi-Omics and Integrative Approach towards Understanding Salinity Tolerance in Rice: A Review

**DOI:** 10.3390/biology11071022

**Published:** 2022-07-07

**Authors:** Pandiyan Muthuramalingam, Rajendran Jeyasri, Kasinathan Rakkammal, Lakkakula Satish, Sasanala Shamili, Adhimoolam Karthikeyan, Alaguvel Valliammai, Arumugam Priya, Anthonymuthu Selvaraj, Pandiyan Gowri, Qiang-Sheng Wu, Shunmugiah Karutha Pandian, Hyunsuk Shin, Jen-Tsung Chen, Venkidasamy Baskar, Muthu Thiruvengadam, Manoharan Akilan, Manikandan Ramesh

**Affiliations:** 1 Department of Biotechnology, Science Campus, Alagappa University, Karaikudi 630 003, India; pandianmuthuramalingam@gmail.com (P.M.); jeyasri8220@gmail.com (R.J.); roczbiot7@gmail.com (K.R.); vallimn1@gmail.com (A.V.); priya6bt@gmail.com (A.P.); selvarajbiotech@gmail.com (A.S.); sk_pandian@rediffmail.com (S.K.P.); 2 Department of Horticultural Science, Gyeongsang National University, Jinju 52725, Korea; 3 Department of GreenBio Science, Gyeongsang National University, Jinju 52725, Korea; 4 Avram and Stella Goldstein-Goren Department of Biotechnology Engineering, Ben-Gurion University of the Negev, Beer Sheva 84105, Israel; lsatish@post.bgu.ac.il; 5 The Jacob Blaustein Institutes for Desert Research, Ben-Gurion University of the Negev, Beer Sheva 84105, Israel; shamili.kanna@gmail.com; 6 Subtropical Horticulture Research Institute, Jeju National University, Jeju 63243, Korea; karthik2373@gmail.com; 7 Department of Botany, Science Campus, Alagappa University, Karaikudi 630 003, India; gowripandi98@gmail.com; 8 College of Horticulture and Gardening, Yangtze University, Jingzhou 434025, China; wuqiangsh@163.com; 9 Department of Chemistry, Faculty of Science, University of Hradec Kralove, 50003 Hradec Kralove, Czech Republic; 10 Department of Life Sciences, National University of Kaohsiung, Kaohsiung 811, Taiwan; jentsung@nuk.edu.tw; 11 Department of Oral and Maxillofaciel Surgery, Saveetha Dental College and Hospitals, Saveetha Institute of Medical and Technical Sciences, Chennai 602 105, India; baskarbt07@gmail.com; 12 Department of Crop Science, College of Sanghuh Life Science, Konkuk University, Seoul 05029, Korea; 13 Department of Plant Breeding and Genetics, Anbil Dharmalingam Agricultural College and Research Institute, Tamil Nadu Agricultural University, Trichy 620 027, India; akilanmkarur@gmail.com

**Keywords:** agricultural practices, bioinformatics, biotechnological tools, breeding, multi-omics, rice, salinity stress, transcription factors

## Abstract

**Simple Summary:**

Rice is one of the most significant food crops worldwide, predominantly in Asian countries. Rice plant growth and yield are profoundly affected by salinity stress. Understanding the mechanisms of salinity stress is highly complicated. Therefore, to understand the molecular mechanisms, enhance the yield and development of salinity tolerant cultivars, and achieve spectacular gains in the future, the use of high-throughput frontier technologies must be knotted with a research basis. Keeping these lacunae in mind, this comprehensive review addresses the literature gap and presents the multi-omics and integrative approaches to harnessing the novel avenues of salinity stress mechanisms in rice. Further, this review will be a significant pioneer for researchers working with plant stress biology by employing multi-omics and integrative approaches.

**Abstract:**

Rice (*Oryza sativa* L.) plants are simultaneously encountered by environmental stressors, most importantly salinity stress. Salinity is the major hurdle that can negatively impact growth and crop yield. Understanding the salt stress and its associated complex trait mechanisms for enhancing salt tolerance in rice plants would ensure future food security. The main aim of this review is to provide insights and impacts of molecular-physiological responses, biochemical alterations, and plant hormonal signal transduction pathways in rice under saline stress. Furthermore, the review highlights the emerging breakthrough in multi-omics and computational biology in identifying the saline stress-responsive candidate genes and transcription factors (TFs). In addition, the review also summarizes the biotechnological tools, genetic engineering, breeding, and agricultural practicing factors that can be implemented to realize the bottlenecks and opportunities to enhance salt tolerance and develop salinity tolerant rice varieties. Future studies pinpointed the augmentation of powerful tools to dissect the salinity stress-related novel players, reveal in-depth mechanisms and ways to incorporate the available literature, and recent advancements to throw more light on salinity responsive transduction pathways in plants. Particularly, this review unravels the whole picture of salinity stress tolerance in rice by expanding knowledge that focuses on molecular aspects.

## 1. Introduction

Plants grown in field conditions are simultaneously exposed to combined abiotic stressors, viz. salinity, drought, heat, high temperature, UV flooding, heavy metals and cold, etc., [1,2,3]. Among these, salinity is one of the predominant abiotic stresses faced by individuals practicing agriculture. In general, salt stress is classified into two types, short and long-term. Short-term stress is alleged by the root system, triggering osmotic stress and also causing a reduced amount of water potential. Long-term stimulates the toxicity of ions owing to the imbalances of cytosolic nutrients [4]. Furthermore, crop productivity is greatly encountered by salinity stress, which also hinders plant growth and yield, restricts land usage, causes nutritional deprivation, ion toxicity, and osmotic stress [4,5]. Salinity stress is also associated with an imbalance of ions, delayed germination, seedling growth, and decreased seed set amounts [6]. The soil salinity issue has been exasperated by environmental changes, drought, and agricultural practices, namely irrigation which affects the global cultivated and maximum of irrigated lands are pretentious by salinity [7]. Hence, enhancing the salinity tolerance is essential in important food crops, particularly rice, with the effective use of soil-saline land. The presence of stress-tolerant genes supports the plant’s survival in extreme climatic conditions and enables sustainable agricultural production.

Rice (*Oryza sativa* L.) is a staple and essential monocarpic cereal, feeding people around the globe, particularly in Asian countries [8]. Annually, around 400 million tons of paddy rice are produced to cater to almost fifty percent of the population in the world [9]. Thus, it is an essential crop for food security which improves livelihoods and poverty alleviation, not only impacting Asia but the entire globe. While the global production of rice has been significantly growing since the 1960s by the Green Revolution, the constantly rising human population and climatic variations are the foremost bottlenecks for sustained production and protection now and in the near future. Further, it is widely expected that the global population will reach around 9 billion by 2050 [10,11]. Besides, environmental stressors negatively constrain plant growth, survival, and production [2,3]. In addition, paddy rice exhibits its unique tolerance and susceptibilities to diverse abiotic stresses, notably, salinity stress among all other cultivated food/cereals [12]. It depends on the rice varieties. For example, the *japonica* rice variety is more susceptible than the *indica* rice variety [13]. The rice plant growth is hindered by the soil salinity by disturbing photosynthesis machinery in plants by weakening the plant photosystems, ion distribution, homeostasis, carbon fixation, and electron transport chain [10,14]. Thus, to enhance the crop yield and productivity and salinity tolerance, it is imperative to investigate the physiochemical mechanisms with their modifications of rice to saline stress. Consequently, the advancement of plant science in developing developed and underdeveloped countries unravel novel avenues for rice production, and consumption is predicted to increase in the future.

These issues alarm to throw more light on plant stress biologists to evaluate the biochemical and molecular stress machinery and their signaling network in rice. Significantly, the available whole rice genome and transcriptome projects [15], multi-omics approaches [16,17], bioinformatic platforms [18], agricultural practicing elements [19,20,21,22], breeding [23], and recent literature pave the way to identify and annotate the stress-responsive key players with their regulatory functions and also provide the deeper molecular insights. These integrated approaches and holistic strategies used for the dissection of dynamic networks and altering the repercussions in plants support them in controlling abiotic stresses, particularly salinity stress. These claims will aid in unraveling the stress tolerance and adaptation to saline and other environments and in developing stress-tolerant plants with enhanced yield.

**Figure 1:**
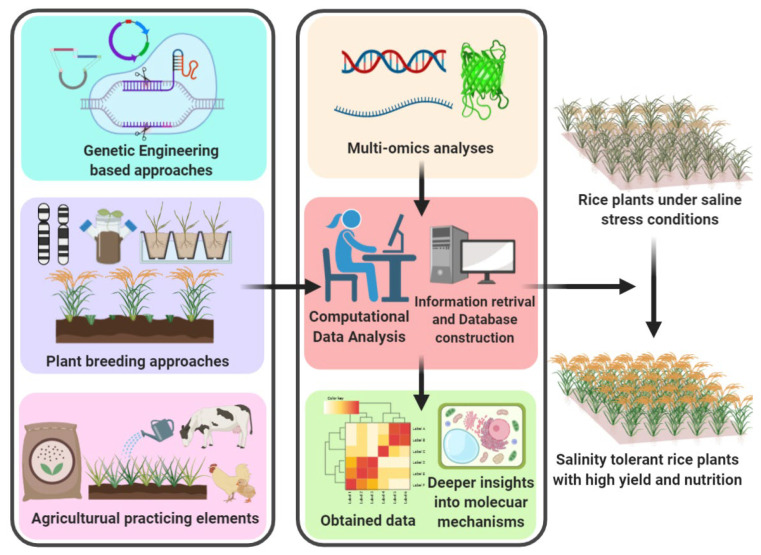
Schematic representation of various biological approaches speculated to develop the salinity tolerant rice plants (the image created in BioRender.com accessed on 2 June 2022).

This review article summarized the diverse biological strategies aimed at overcoming salinity stress in rice plants (Figure 1). Salinity stress mechanisms are delineated in connection with genetic modifications to enhance salinity tolerance in different ways. In addition, stress-responsive gene identification through multi-omics tools is essential for elucidating the tolerance/avoidance mechanisms described, and it aims to fit diverse reports related to determinants of salt tolerance, molecular genetic improvements, and functionalities into a proposed pathway network. Furthermore, agricultural practicing elements are also used to improve salinity tolerance. The main goal is not merely to collate the facts but to extend the novel ideas about salinity tolerant analyses in the hope of introducing new avenues in this field.

## 2. Influence of Salinity Stress in Plants

**Figure 2:**
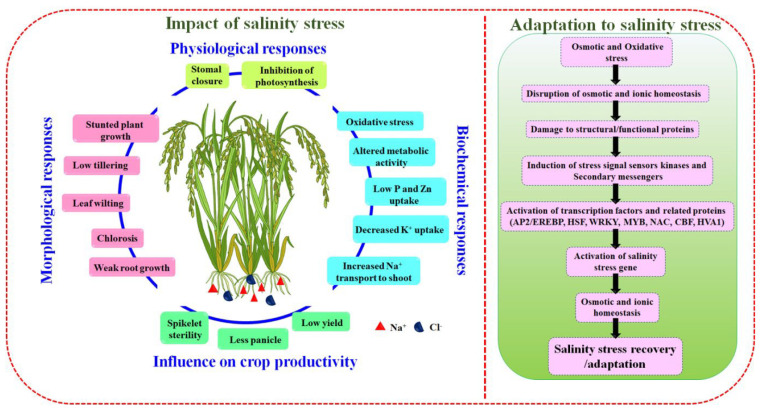
Impact and adaptation of salinity stress in rice.

Among the various abiotic/environmental stressors, saline soil is considered predominant as it can negatively impact crop productivity [4]. The crop plants can be classified into two types, halophyte (salt-tolerant) and glycophyte (salt-sensitive), based on the salinity stress tolerant level. Approximately 50% of the arable land is imputed to become saline in the year 2050 [24]. Furthermore, salinity stress commonly induces two types of responses to plant growth: (1) ion-excess or salinity-specific [ionic stress] and (2) water-deficit or osmotic effect [osmotic stress] [25]. Salinity-specific or ion-excess effect is attributable to the intake of the excess level of Na^+^ and Cl^−^ in the transpiration stream, which leads to severe cell injuries, further reducing the growth. However, the osmotic or water-deficit effect is caused by excess saline concentration in the soil, which reduces water uptake of plants, resulting in a reduction in transpiration and growth rates (Figure 2) [26,27].

Saline stressors are not only stimulated by salinity stress but also induce drought tolerance because these stressors are intimately related. Drought (water deficit) condition is also caused by dissolved salts in the soil as they affect water potential. The important machinery and common effects of salinity and water deficit stress are transcription factors (TFs). TFs with their family members are involved in drought stress signaling pathways that overlap with the salinity stress-associated signal transduction pathways [28]. The effect of salinity stress has drastically affected the plants regarding leaf expansion, protein biosynthesis, photosynthesis, energy, lipid metabolisms, and other biosynthetic and metabolic processes [29].

Besides, salinity stress indirectly affects the development of plant growth by deteriorating the rate of stomatal conductance and photosynthesis [30]. The reduction rate of the stomatal aperture is contemplated as a conspicuous response that ensues in a nutshell after the plants encounter salt stress. Stomatal conductance responses are undeniably stimulated by the osmotic consequence of the salinity from outside of the roots [29]. In addition to the effect on stomatal opening, salinity stress affects the density and size of the stomata, which further leads to a reduction in stomatal conductance. Subsequently, photosynthesis (CO_2_ uptake) and transpiration rates (i.e., water loss) are also decreased. Besides, the reduced rate of photosynthetic process upsurges the build-up of ROS (reactive oxygen species) and diverse antioxidant activities, including peroxidises catalase (CAT) and superoxide dismutase (SOD) [31,32]. Diverse saline stress mechanisms have been proposed in plants, including stimulating the regulation of salt-tolerant genes and exclusion of salinity from the soils and plants. In addition, the accumulation of osmoprotectants is the most important factor that maintains the plasma membrane integrity, scavenging the free radicals and defending the plant cells from salinity stress conditions (Figure 2) [1,33,34]. On the whole, such analyses delineated the impact of salinity stress on diverse plants in accordance with the soil saline concentrations. Understanding the osmotic and ionic stress mechanisms is an alternative path to developing salt-resistant plants. In addition, these studies also suggest the identification of salt-tolerant/responsible genes which enables plants to survive and withstand saline stress conditions.

### 2.1. Saline Stress and Their Physiochemical Responses in Rice

Salinity stress responses in a plant are a highly complex phenomenon, and it can acclimatize to salinity stress by modifying the various developmental and biochemical, molecular-physiological processes. Under salinity stress conditions, plant responses were separated into two main categories: initial phase, ion-independent response, which occurs in minutes to days to cause a negative effect on stomatal closure and inhibits the expansion of shoot cells [28]. The second phase is an ion-dependent response that occurs in days and/or weeks to build up the ion cytotoxicity, which downturn the premature leaf senescence, metabolic processes, and cell death [28,35]. Generally, in plants, stomata are essential for controlling the gas exchanges; salinity stress severely affects the stomatal opening, size, and density. Furthermore, salinity stress could also affect the overall developmental mechanism of the plant by demonizing the level of stomatal conductance and photosynthesis [36]. In the meantime, the rate of transpiration and photosynthesis processes are also drastically decreased. When compared to other cereals, rice is more susceptible to saline stress [37,38]. These studies state that alterations in the physiological and biochemical mechanisms are interlinked to the resistance of rice plants under salinity conditions.

In soil, salt enters the root system via the symplastic and apoplastic pathway, which stimulates several signaling cascades that activates the ionic tolerance by limiting the influx of Na^+^ into the root and reducing Na^+^ translocation [28,39]. Direct transcriptional flow occurs in the apoplastic pathway, and this flow continues betwixt the outside cellular membrane and xylem [28]. In addition to the same trajectory, the apoplast comprises cell walls, extracellular spaces, xylem, phloem, and tracheids. In rice, the apoplastic pathway could be responsible for the absorption and translocation of half of the total Na^+^ and Cl^−^ [40,41]. At the same time, the symplastic pathway interconnects the cell via the cytoplasmic channel. In this pathway, salt molecules move through the plasmodesmata region in the cells. The total uptake of Na^+^ and Cl^−^ into Arabidopsis roots via this pathway is catalyzed by specific transporters. Previous studies indicate the wide variety of distinct mechanisms with their involvement, and hence the physiological impact is far from clear. NSCCs (Non-selective Cation Channels) represent the two different gene families. One is CNGCs (Cyclic nucleotide-gated channels), and the second one is GLRs (Glutamate receptor-like channels) and blocked by Ca^2+^ [42]. The root cell in the apoplastic pathway contains Ca^2+^ concentration in the region of 0.2–0.4 mM, which is as much as necessary to decrease the level by 30–50% in NSCCs mediated flux [43]. Further, extant flux can be reduced by Gd^3+^, La^3+^ and cyclic GMP [28]. In dicot plants like *Arabidopsis*, internal sodium flux was carried via NSCCs genetic. Their closeness to the respective channels is difficult to understand, and their putative role has not been delineated. However, to determine the effect of the apoplastic pathway in rice, thorough more experimental analyses are still required [28,44]. In comparison, monocots have several HKT isoforms different from the Arabidopsis, specifically carrying the selective Na^+^, subclass 1, *AtHKT*1. *OsHKT2*;1 catalyzes in rice plants having the Na^+^ absorption in low K^+^, low sodium environments, and *AtHKT1* functions with distance transport machinery of Na^+^ via phloem and xylem [45].

### 2.2. Ion Toxicity and Ion Homeostasis

The toxic salt effect is specifically correlated to the concentrations of ion molecules, and it may persist in all the cellular compartments, specifically in the cytoplast. Therefore, accurate Na^+^ and Cl^−^ cell measurements are significant to proper toxicity analyses, but these tests are only carried out for comparatively few plants [46]. The interactions between mineral nutrition and salinity are complicated, and they can influence the supply of nutrients and their transportation. Key factors of ion homeostasis induce nutrient imbalances through the rivalry between Cl^−^ and Na^+^ with the nutrient ions. In addition, Fe^2+^, Ca^2+^, K^+^, and Mg^2+^ can affect the overaccumulation of Na^+^ and Cl^−^ in the plant [47]. To increase the quality and nutrient consumption performance of agricultural soils, comprehensive knowledge of the interplay between mineral ions and salinity is of great importance. In rice, cultivars with their changed dispensation of macro- and micro-nutrients and ionic imbalances were observed in various sections of rice during a stress environment [48]. K^+^ is known to be the primary regulatory element factor involved in various metabolic processes under saline stress conditions in important crop plants via elevation of Na^+^ exclusion and osmotic dynamisms. The relation of optimum K^+^ and Na^+^ involves in the salt tolerance mechanism. In addition, the comparative analyses of the K^+^ and Na^+^ ratio in salt-tolerant (IR632 and IR651) and sensitive (IR29) rice varieties and the ratio of K^+^/Na^+^ were lower in sensitive IR29 compared to IR632 and IR651. These results revealed that the variations between the ratio of K^+^ and Na^+^ in rice exhibit the elimination of leaf tissues from Na^+^, and it seems to be a significant factor of saline tolerance/resistance in rice through preservation of the optimum ratio of K^+^ and Na^+^ [49]. Similarly, Reddy et al. (2017) [50] found that the K^+^/Na^+^ ratio played a significant role in rice biomass. The higher ratio of K^+^/Na^+^ showed that reduction in biomass compared to a lower K^+^/Na^+^ ratio. The absorption of Zn, Ca, and P is reduced under saline stress, which is an essential micronutrient as well as the major component of plant enzymes. Although the interaction between K^+^/Na^+^ under salt stress causes Fe homeostasis in rice plants due to uptake of Fe, it was reduced by restricted releases of phytosiderophores which influence the morphology of the shoot and root, including biomass.

### 2.3. Oxidative Stress and Antioxidant Defence Response

The elevated production of ROS molecules in plants is the typical implication of salt stress. Plants exposed to higher salinity condition triggers stomatal closure resulting in a decreased level of CO_2_ availability and fixation. The reduction in the emission of CO_2_ in the C_3_ cycle (Calvin cycle) induces a state of molecular machinery; exposure of chloroplasts and the photosynthetic mechanism of electron transport are sequentially disrupted. Hence, the salt stress induces the increased production of ROS molecules, which include (O_2_^•−^) superoxide, (OH^•−^) hydroxyl radicals, (^1^O_2_) singlet oxygen, and (H_2_O_2_) hydrogen peroxide molecules resulting in oxidative stress [34,48,51]. Generally, ROS molecules are constantly produced in every intracellular organelle, such as chloroplast, peroxisome, and mitochondria in plants by the aerobic metabolism. Furthermore, CO_2_ and O_2_ ratio reduction in chloroplast cells elevate the synthesis of ROS. The well-designed equilibrium between the production of ROS and scavenging mechanisms is disturbed by salt stress which interferes with the plant metabolic activity by causing oxidation of bio-macromolecules, namely protein, DNA, RNA, and lipid peroxidation [52]. Nevertheless, all the plants have efficient enzymatic antioxidant and non-enzymatic antioxidant systems, which detoxify the ROS molecules to defend the plants from oxidative stress. It comprises SOD, CAT, APX (ascorbate peroxidase), GST (glutathione S-transferase), MDHAR (monodehydroascorbate reductase), GSH (glutathione), AsA (ascorbate), tocopherol, and carotenoids [34,53,54,55].

Antioxidant activities in plants were reported to significantly correlate with reduced oxidative and improved saline stress. In rice, it is based on the regulatory function of various metabolites and enzymes of the antioxidant protection machinery to reduce ROS-induced oxidative stress response during saline stress [56,57]. Initial protection is provided by SOD, which instigates the conversion of toxic O_2_^•−^ radical to form less toxic H_2_O_2_ and O_2_. The various researchers assessed the relationship between SOD and H_2_O_2_ accumulation. Improving the adaptive stress response of plants at both physiological and metabolic levels requires increased SOD activity [58,59]. Analyzing the impact of SOD sensitivity to and the resistance of rice genotypes to salt, the results suggested that increased SOD activity is found in tolerant and susceptible genotypes after salt stress [60,61,62]. However, some of the salt-sensitive rice cultivars revealed decreased SOD activity; it may reduce the metabolic activity to delimit oxidative stress creating an imbalance of redox potential and cell death in rice [63,64]. Generally, rice isoforms of 8 SOD genes showed differential expression signatures in salinity-resistant and susceptible rice varieties. The variations of SOD isoforms and their responses in salt-treated plants may be correlated with the localization of subcellular organelles [65,66]. After activation of the stress signaling pathway, an excess amount of H_2_O_2_ molecules is scavenged by the enzymatic and non-enzymatic antioxidants such as APX, CAT, AsA, glutathione reductase (GR), and guaiacol peroxidase (G-POD), etc. Compared to other antioxidant enzymes, CAT effectively scavenges the H_2_O_2_ under saline stress conditions. The affinity of the CAT enzyme is (mM) very less, but activity is crucial in a higher level of ROS. The increased CAT activity was observed in GIZA 178, Pokkali, Nonabokra, and CSR10 [67,68]. In addition, Vighi et al. (2017) [62] reported on the correlation between CAT isoform and CAT in salt-tolerant rice genotypes, which indicates a direct connection between the CAT activity and transcript levels may be inhibited by post-transcriptional/translational dynamisms. Under salinity stress conditions, the CAT isoforms are highly expressed in various plant parts to elevate the CAT activity [69,70].

Rice plants maintain a high APX level via the AsA-GSH cycle. Here, AsA is acted as a hydrogen donor by APX and converts the H_2_O_2_ to water and oxygen. APX has a strong affinity towards H_2_O_2_ and enables redox homeostasis, thereby inducing the reactions of salt stress by decreasing the ROS toxic effect [71]. Isoforms of APX genes played a significant role in enhancing salinity resistance [72,73]. During salt instigates, the oxidative stress mechanism, peroxidase (POD), plays a vital part in rice stress tolerance and acts as a biomarker. The elevated POD level is directly linked with the higher expression of the players involved. Two different rice variants with salt tolerance showed a negative effect betwixt POD activity and saline resistance [74]. Increased POD activity in salt-sensitive rice cultivars plays a predominant function in the development of root lignin synthesis and also renders it to water loss [70]. However, the synchronized activity of SOD along with other antioxidant enzymes, including CAT, POD, and APX, is highly required for ROS molecules scavenging activity in rice during salt stress. To protect against the modifications of salt instigated oxidative stress, antioxidant plays a prominent role in salinity tolerance in rice.

GR is a significant enzyme that catalyzes the GSSG (oxidized glutathione disulfide) toward GSH to maintain a ratio of GSH/GSSG during a salinity stress environment. During salinity stress, isoforms of GR expression were induced higher in tolerant cultivar when compared to a susceptible variety [75]. In addition, the elevated redox concentration of antioxidants AsA/DHA (dehydroascorbic acid) and GSSG enhanced the stress resistance, thereby reducing the ROS accumulation and causing a notable effect on plant differentiation that strengthened the salt tolerance to rice plants [76]. The enhanced MDHAR, GR, and DHAR (dehydroascorbate reductase) activities in *O. sativa* elevate the transformation of AsA and GSH, enhancing the GSH/GSSG/DHA cellular redox ratio in salt-stress treated rice plants. On the whole, unraveling the oxidative stress and antioxidant defense responsive enzyme associated with candidate genes with their regulatory mechanisms enable us to develop the salt-tolerant rice genotypes.

### 2.4. Salinity Stress-Responsive Genes and TFs in Response to Salinity Stress

Salinity stress responses are highly complex and interconnected and involve a plethora of genes. cDNA microarrays have become a more efficacious tool for throughput experimental analyses of abiotic stress encoding players, notably salinity stress-associated candidates. Salinity-responsive genes have been classified based on their function, such as transporters, carbohydrates, protein metabolism, energy metabolism, hormones, reactive oxygen network components, osmoprotectants, cell walls, and signal transduction components [34,77]. In rice and Arabidopsis, genome-wide scale microarrays harboring an almost complete set of transcript sequences have been produced in accordance with the availability of whole-genome sequence (WGS) [15,78] and retrieval of a complete set of cDNA sequences [79]. In rice, 1700 independent cDNAs microarrays derived from high salinity, drought, and cold treated rice plants were used to identify 57 salinity-inducible genes. Among them, 15 showed responses to drought, cold, and ABA treatments through northern blot analysis [80]. Another study revealed a microarray consisting of ~9000 unique genes to identify 486 salt stress-responsive genes in shoots of salinity tolerant variety, Nona Bokra (*O. sativa* L. ssp. *indica* pv. Nona), compared to untreated control [81]. In transgenic plants, microarray experiments showed that TFs regulate the salinity of stress-responsive genes. Primarily, the member of DREB1/CBF, DREB1A/CBF3 TFs which target several well-characterized stress-tolerance candidate genes, including osmoprotectant biosynthesis protein, late embryo abundant (LEA) proteins, RNA-binding proteins, and so on [82,83]. A glimpse of representative key players in response to abiotic stresses, including salinity stress and their functions, were described in Supplementary Table S1.

TFs are the essential players that act in association with several transcriptional regulators and are involved in transcriptional reprogramming, chromatin structure remodeling, or altering the biomolecule, namely proteins. TFs play a prominent role in plant stress signaling and transduce the various signal transduction pathways. In addition, TFs interacting with the *cis*-regulatory elements were present in the promoter regions of stress-responsible genes, which have differentially regulated the expression of several downstream genes exhibiting the enhancement of abiotic stress tolerance [17,84]. So far, approximately 58 TF families have been categorized [85]. NAC, MYB, bHLH, bZIP, WRKY, HSF, ZF-HD, ABF, AP2-EREBP, and DREB are the significant TFs superfamilies. These TF family members modulate the cellular and physiological processes, viz. cellular differentiation, plant development, seed storage, callus formation, somatic embryogenesis, seed development, biosynthesis of secondary metabolites, and control the plant cell metabolisms [17,84,86,87]. Furthermore, the mentioned TFs also play a major role in various abiotic stress mechanisms such as cold, submergence, drought, UV, temperature, desiccation, heavy metals, wounding, osmotic stress, and particularly salinity stress [17,84,88,89,90,91]. These TFs and their functional role and abiotic stress regulation, mainly salinity stress, are summarized in Supplementary Table S2. Moreover, these characterized regulons and a large spectrum of other TFs are also involved in abiotic stresses, including salt stress response, thus accelerating the essential role in plant stress endurance. Hence, these diverse stress-associated TFs independently do their function, unquestionably enabling the molecular level interactions to exit betwixt them. In addition, further study is needed to identify novel salt stress-tolerant TF family members and unravel the regulatory avenues in rice.

## 3. Role of Plant Hormones and ABA-Mediated Responses in Salinity Stress

### 3.1. Plant Hormone Signal Transduction Pathways

In plants, various signal transduction pathways and cross-talks work together under severe biotic or abiotic stress conditions. These responses are mediated by the interactions of several plant hormones [92]. Plant growth and development, cellular differentiation, and cellular metabolisms are regulated and controlled by the phytohormones. It also plays a vital role in recognizing the signal from various environmental conditions such as salinity, drought, osmotic, cold and heavy metal, etc. [93]. There are nine important phytohormones available; auxins (AUX), ABA, cytokinins (CK), brassinosteroids (BRs), ethylene (ET), gibberellins (GA), jasmonic acid (JA), salicylic acid (SA), and strigolactones (SL) have been tangled in diverse plant differentiation processes and to abiotic stresses adaptive responses [33,94]. Earlier studies reported that a few signaling molecules from the light and plant hormone pathways allow the photoreceptors to affect plant growth and yield. In addition, the output of plant hormone signaling involves alterations in the expression dynamism of several candidate players, many of which act as a decisive response in cell expansion and division [95].

For example, phytohormones derived from polyunsaturated fatty acids, namely jasmonates (JA, its precursors and derivatives), are involved in biotic and abiotic stresses, including salinity stress conditions. It is an important immunity hormone involved in various signal transduction pathways, along with gene networks, regulatory proteins, signaling intermediates, catalytic enzymes, and other biomolecules which defend the cells from the harmful effects of abiotic stress [33]. In response to abiotic stress conditions, a higher accumulation of JA activates the defense responses, mainly protecting plant cells against salt stress. In connection with that, it mainly involves the antioxidant enzyme and other defensive compounds [94]. We understood the JAs biosynthesis and signal transduction pathways play a vital role in defense and resistance under abiotic stress conditions in rice [94].

Phytohormone signaling pathways affect plant growth and regulation. When considering the effect of gene expression, concentration and perception signaling are the central issues [96]. On the whole, we focus the signal transduction and biosynthesis pathways of phytohormones with their role in response to salinity stress. Further analysis will also explore the underlying actions of plant hormonal pathways and their interactions to unravel the avenues between abiotic stresses and growth-related processes.

### 3.2. ABA-Dependent and Independent Pathway

Abiotic stresses, particularly salinity, drought stress, toxicity, nutrient insufficiency, and flooding, restrict agricultural productivity globally [97]. Plant development in saline environments is hampered by the action of ions on metabolism or by damaging groundwater interactions [98]. Plant hormones are dynamic signal compounds in plant species that are biologically produced and are associated with the development of plant hormonal levels, including through drought or excessive soil and water saline [99]. Under abiotic stress conditions, phytohormone(s) levels are significantly regulated and reduce plant growth and metabolisms [98]. Out of the nine well-known plant hormones, ABA, ET, JA, and SA are considered notable stress-responsive hormones. In particular, ABA is an essential hormone that regulates plant growth, reproduction, stress responses, and metabolism. It is involved in various physiological and molecular functions in plants, including adaptive responses, seed germination, leaf senescence, stomatal closure, cuticular wax depositions, dormancy of the flower buds, osmotic control, and plant growth inhibition. Furthermore, ABA regulates downstream responsiveness to biotic and abiotic factors that cause changes in the environment via transcriptional and post-transcriptional pathways [100]. In addition, ABA and their biosynthetic key players in plants are rapidly elevated by abiotic stresses, including salinity stress [33]. ABA is among the most important stress-responsive endogenous hormones, and it plays an important part in the salt stress defense system [100,101]. ABA is a central integrator that links and reprograms the complex developmental processes and salt stress in plants, especially osmotic stress and adaptive signaling cascades [102,103]. Salinity stress enhanced the content of endogenous ABA in leaf tissues of *Brassica* sp. *Phaseolus* sp. [104,105] and *Zea mays* [106] showed a substantial correlation with growth inhibition [98].

In plants, ABA is synthesized in the root system and transmitted via vascular tissues, where it promotes stomatal closing signals in a range of cell types, including guard cells. Plants’ adaptive responses to diverse stress situations may be split into two routes: ABA-dependent and ABA-independent, and these pathways govern the osmotic stress-responsive expression of genes [107]. ABA signal transduction in cotton plants is mediated by both ABA-dependent and -independent mechanisms [108].

**Figure 3:**
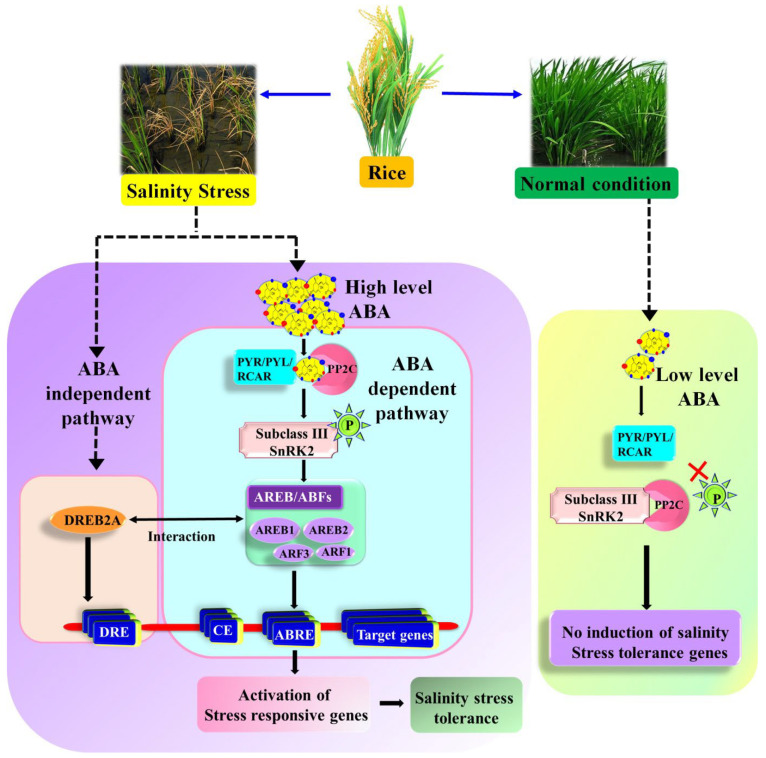
ABA-dependent and -independent pathway in rice under normal and salinity stress conditions. ABA-dependent pathway includes regulatory components (PYR/PYL/RCAR), PP2C, subclass III SnRK2, and four ARBB/ABF transcription factors such as AREB1, AREB2, ARF3, and ARF1 gene expression. ABA-independent pathway includes transcription factors (DREB2A) and stress-related genes. CE—coupling element.

Under normal conditions, ABA levels fall, and the activity of sucrose nonfermenting 1-related protein kinases (SnRK2s) is suppressed with PP2C phosphatases, resulting in dephosphorylation. Due to salinity stress, in the ABA-dependent pathway, increased ABA levels bind to its receptors such as PYR/PYL/RCARs, leading to the inactivation of PP2Cs activity. The SnRK2s activated by dissociating from the PP2Cs lead to phosphorylation of downstream targets and activate the ABA-induced molecular and physiological responses [108,109]. In ABA-dependent gene expression, a collection of TFs, a *cis*-acting component, an ABA-responsive element (ABRE), and ABRE binding factors (AREB/ABFs) play a significant role (Figure 3). High salinity, drought, and ABA increase the expression of AREB1 or AREB2/ABF2 or ABF4 and ABF3 factors in vegetative tissues. AREB/ABF TFs are triggered in the ABA-dependent system, and ABA-responsive gene expression is controlled by SnRK2s via phosphorylation of AREB/ABFs during osmotic stress [107,110].

Similarly, in addition to osmotic stress, a *cis*-element, dehydration-responsive element/C-repeat (DRE/CRT), and its binding protein 2 (DREB2) TFs are required for the ABA-independent gene regulation [107]. The DREB2 protein acts as plant-specific TFs and members of the AP2/ERF family (Figure 3). In *Arabidopsis*, DREB2A and 2B are highly stimulated by abiotic stresses, notably high saline, heat, and drought stress [111]. Complete transcriptome and metabolome investigations in *Arabidopsis* and *O. sativa* have revealed the role of ABA-dependent and ABA-independent pathways signaling in metabolic changes under osmotic stress treatments. [112]. Under normal conditions, transcriptional and protein levels of DREB2A, an important TF in the ABA-independent mechanism, are regulated through GRF7 and DRIPs. Whereas AREB/ABF and DREB2A expression is controlled by the subclass III SnRK2s (Figure 3), these kinases serve as a link in the pathway that connects ABA-dependent and ABA-independent gene expression [107].

To enhance drought resistance, ABA controls numerous stress-related genes in cotton plants. In Arabidopsis, overexpression of the ABA-induced cotton gene *GhCBF3* improved salinity and drought resistance in transgenic lines, with increased proline, leaf water, and chlorophyll content in transgenic lines when related to wild type. Expression levels of AREB1 and AREB2 were increased, and stomatal aperture was reduced in transgenic lines than in wild type due to the presence of ABA. We deduced that *GhCBF3* improves drought and osmotic stress via the ABA signaling pathway [108].

ABA is also required for SL-mediated crop salt tolerance. During salt stress, ABA concentration in mycorrhiza-colonized lettuce roots, regulated via salt-induced SL synthesis, leads to activation of the ABA biosynthetic gene *LsNCED2* [103]. ATAF1, ATAF2, AREB/ABF, CUC, DREB, MYC/MYB, and NAM, and other *cis*-acting factors ABRE, DRE, MYCRS/MYBRS, and NACRS are all engaged for both ABA-dependent and ABA-independent transcriptional regulation ranging from stress signal sensitivity to gene expression [113]. ABA levels increased due to high salinity stress leads to significant changes in gene expression and adaptive physiological responses [114]. ABA increases stomatal closure to minimize water evaporation and modulates stress-related expression levels, reducing the effects [115]. In *Arabidopsis* and *O. sativa*, high salinity-inducible (osmotic responsive) genes are activated due to the presence of ABA [80,116,117]. This work suggests that the participation of ABA-dependent and ABA-independent signal transduction pathways may be important for salt stress resistance in *O. sativa*.

## 4. Breeding as a Key Tool for Salinity Tolerance in Rice

Breeding techniques are an efficient and cost-effective way of generating rice cultivars to grow successfully in saline environments. The primary goal of rice breeding programs for salinity resistance is to enhance the respective alleles involved in the salinity tolerance in rice. Assessing the physiological traits is the best way to refine the salinity tolerant genotypes. However, physiological traits are regulated through many genes, which contribute to the continuous difference. Salinity stress affects flowering and yield traits, including spikelet number per panicle, panicle length, and grain yield, and also delays the emergence of the panicle. Rice exhibits diverse levels of salinity resistance mechanisms at various growth stages [118,119]. Hence, salinity breeding programs attempted to screen the segregating populations in the seedling stage and examine the potential of salinity tolerant genotypes from the primary screening results at the reproductive stage, likely during the field conditions [120]. In breeding programs, conventional methods are extensively used to enhance the salinity tolerance in rice, including; (i) screening of germplasms for salinity tolerance in precise environments, (ii) utilizing the genetic variation that already exists in the available genotypes through pedigree or backcross breeding methods, (iii) producing hybrids by elite parental lines resulted from large scale germplasm screening, (iv) interspecific hybridization; transfer the traits linked to the salinity tolerance from wild relatives into popular rice cultivars to raise the tolerance level, (v) mutation breeding; exploiting the variations generated by induced mutations [121,122,123,124]. By exploring conventional breeding methods, considerable success has been achieved in enhancing salinity tolerance. Several tolerant cultivars were developed and commercialized in various countries by exploiting the existing salt tolerance, extensively used sources including Pokkali, Kalarata, and Nona-Bokra and rice salt-tolerant wild relatives [125]. Of these, Pokkali is highly tolerant compared to other tolerant genotypes, and therefore it is used as a promising donor for salinity tolerance breeding programs. However, efforts to enhance salinity tolerance by traditional breeding strategies have experienced problems, and progress is slow. Several researchers highlight numerous points for the slow development in breeding salinity tolerance, such as poor knowledge of salinity and environmental interactions, the complexity of genetic and physiological mechanisms, salinity tolerance controlled by minor or polygenes, low heritability, and also the low-level expression of the salt tolerance traits [23,126].

## 5. Genome-Based Breeding Approaches to Alleviate Salt Tolerance in Rice

The drawbacks of breeding techniques are reduced by using rice genomic resources and tools for precise genomics-assisted salinity breeding. In particular, the application of molecular markers to investigate the QTLs linked to the salinity tolerance mainly in discovering precise regions/loci of chromosomes that will assist in increasing the efficacy of selection in the breeding programs [127]. Second-generation sequencing technologies permit the mass sequencing of genomes and transcriptomes through cost-effective and straightforward sequencing platforms that generate a vast array of genomic information. Thus, genomic resources are meaningfully well defined in rice. The two subspecies, *japonica* (cv. Nipponbare) and *indica* (cv. 93–11), genomes, organelle genomes (Chloroplast and mitochondrial genomes), and ten different wild rice genomes are being sequenced [128]. All the sequence data are deposited and freely accessible in the public domain. Therefore, MAB (marker-assisted breeding) in rice has become more standard, and many SSR and SNP markers are available for rice breeding programs [129,130,131]. Traditional map-based cloning, GWAS (genome-wide association studies), and MARS (marker-aided recurrent selection) are the various marker-based strategies now followed for the mapping and introgression of QTL/gene (s) for salinity tolerance in rice. Multi-parent advanced generation intercross (MAGIC) populations are more useful for trace the QTL/gene(s) [132]. GWAS of the MAGIC population revealed a novel QTL for salinity tolerance in rice [133]. Moreover, coupling with recent advances, including gene editing, high-throughput genotyping, genomic selection (GS), and marker-assisted selection (MAS), with speed breeding (SB), accelerated crop improvement [134]. Rana et al. [135] demonstrated that the SB combined with SNP-based MAS provides a fast and efficient approach to enhancing the salinity tolerance in rice. The introduction of hst1 (*OsRR22*) from “Kaijin” into high-yielding “Yukinko-mai” resulted in line “YNU31-2-4”, adapted to salinity stress at the vegetative and reproductive stages with better yield because of improved photosynthesis, ion homeostasis, regulation of Na^+^ uptake, and xylem loading of Na^+^ to shoot. Besides, several potential players linked with salinity tolerance were identified by comparing the transcriptomic and proteomic datasets of rice under salinity stress vs. control conditions or tolerant vs. susceptible genotypes [136,137].

### QTLs and Candidate Genes for Salinity Tolerance in Rice

Many QTLs and candidate players have been reported for the salt tolerance traits of rice. Bonilla et al. (2002) [138] identified one of the major salt tolerance QTLs *Saltol* on chromosome 1, using a RIL population of Pokkali (tolerant) × IR29 (susceptible). The *Saltol* QTL shows a variation of about 64.3–80.2% in shoot Na^+^/K^+^ ratio and is considered a potential QTL for MAS (marker-assisted selection) of salinity tolerance in rice [139]. In another research, QTL analysis of F_2:3_ populations derived from a cross between *indica* cv. Nona-Bokra (tolerant) and *japonica* cv. Koshihikari (susceptible) detected two major QTLs, *qSKC-1* and *qSNC-7*, for shoot K^+^ concentration. *qSKC-1* explaining a 48.5% variation in chromosome 1, is interlinked with shoot K^+^ ion concentration, and *qSNC-7* explaining 40.1% of the variations in chromosome 7, is associated with shoot Na^+^ concentration [140]. Subsequent cloning of the *qSKC-1* locus revealed that it represents a sodium transporter that helps drive the K^+^ homeostasis during salinity stress through unloading Na^+^ from the xylem [141]. Notably, Lee et al. (2006) [142] mapped QTLs (*qST1* and *qST3*) conferring salt tolerance at the early seedling stage on chromosomes 1 and 4 from the cross betwixt Miyang 23 (*japonica*/*indica*) and Gihobyeo (*japonica*). QTL *qST1* was flanked by the closely linked markers Est 1–2 and RZ569A with the phenotypic variation of 27.8%, whereas *RG179* and *RZ596* flanked *qST3* explained 9.1% of the variation. Ammar et al. (2009) [143] detected 25 major QTLs for salinity tolerance traits, each QTL showing >10% of phenotypic changes, were localized on chromosomes 1, 2, 3, and 8. These QTLs were imputed by the F_2_ population and came to light from a cross between CSR27 (tolerant) and MI48 (susceptible). The further investigation involved the same population by integrating genetic mapping and bulked transcriptome expression profiling conducted by Pandit et al. (2010) [144], resulting in the possible salt-responsive genes. A total of eight important QTLs associated with salt ion concentrations were localized on chromosomes 1, 8, and 12. Notably, the QTL conferring stress sensitivity index for spikelet fertility was co-located on chromosome 8. Fifteen QTLs associated with salt tolerance traits (root dry weight, shoot dry weight, total dry weight, and salinity tolerance score) were located on chromosomes 1, 2, 3, 6, 7, 9, and 10, with 8–26% of the phenotypic variation were obtained in 87 introgression lines developed from Teqing (*indica* cv.) and the accession of wild type rice (*O. rufipogon*). Of these, 13 QTLs from *O. rufipogon*-derived alleles contribute to the salinity resistance in the Teqing background [145]. Similarly, two sets of balanced introgression lines generated from a cross between both tolerant *japonica* variety, Xiushui09, and susceptible *indica* line, IR2061–520–6-9, were used to detect the QTLs controlling salinity tolerance; a total of 47 QTLs, including 26 main-effect QTLs and 21 epistatic QTLs. Of these, several QTLs mainly contribute the salinity tolerance and are also essential for salinity tolerance breeding [146].

In another study, sixteen QTLs explaining 4–47% of the phenotypic variation at the reproductive stage were found on chromosomes 1, 7, 8, and 10 using the F_2_ population derived from Cheriviruppu (tolerant) and PB1 (susceptible). The maximum amount of QTL clusters for diverse component traits was co-localized on chromosomes 1 and 7 [147]. Zheng et al. (2015) [148] identified QTLs encoding with salt tolerance traits in accordance with the linkage and association mapping. In this study, Changbai 10 (tolerant) and Dongnong425 (susceptible) were used to develop mapping population and QTL analysis. It revealed that 13 QTLs, including two QTLs for visual tolerance score, four QTLs for concentrations of Na^+^ in shoots, three QTLs for concentrations of K^+^ in shoots, three QTLs for concentrations of Na^+^ roots, and one QTL for concentrations of Na^+^ roots. On the other hand, using 347 *japonica* accessions, 24 significant marker-trait associations (*p* ≤ 0.01) linking 20 markers were detected through association mapping. Notably, 10 of the SSR markers confirmed the genomic regions for salt tolerance stated in linkage mapping, including six QTLs identified during the QTL analysis. One hundred F_5_ RILs of At354 × Bg352 used for QTL analysis revealed six QTLs in chromosomes 1 and 4, namely, *qSSI1*, *qSL1*, *qSNK1*, *qSL4*, *qSNK4*, and *qSSI4*, explaining 10–16% of the phenotypic variations. All the QTLs contributed by the At354 (tolerant) allele favor salinity tolerance [149]. A total of 285 introgressed genotypes/lines were acquired from the *O. rufipogon* accession (tolerant) and *O. sativa* ssp., *indica* cv. 93–11 (susceptible) evaluated at the seedling stage and used for QTL analysis. A total of 10 QTLs linked with salt resistance were detected on chromosomes 1, 5, 7, 9, 10, 11, and 12, with individual QTLs explaining 2–8% of phenotypic variance. Further RNA sequencing analysis revealed that four candidate genes (Os05g31254, Os05g31620, Os05g32070, and Os10g34730) were co-localized with QTLs for salinity tolerance [150]. He et al. 2019 [151] mapped one major QTL *qSE3* for seed germination and establishment of seedlings under stress conditions. Map-based cloning of *qSE3* exhibited that it encodes a K^+^ transporter gene, *OsHAK21.* Recently, F_2_ populations derived from Dianjingyou 1 (susceptible) and *indica* rice Sea Rice 86 (SR86) (tolerant) detected a 2.78 Mb candidate region (*qST1.1*) associated with salinity tolerance on chromosome 1 [152].

Apart from the traditional QTL mapping studies for salinity tolerance, GWAS was successfully used to detect many loci responsible for salinity tolerance in rice. Kumar et al. [153] used 220 rice accessions to perform a GWAS of 12 various salinity tolerance-related attributes at the reproduction stage. Under saline stress, GWAS identified 20 SNPs linked with the Na^+^/K^+^ ratio and 44 SNPs linked with other traits. In another study, GWAS analysis of 708 rice accessions unveiled the genes related to salinity tolerance and seven genotypes with favorable haplotypes of four candidates associated with grain yield under salinity stress [154]. Yuan et al. [155] conducted a GWAS analysis with 664 cultivated rice accession and identified 21 salinity tolerant QTLs and two candidate genes. Lekklar et al. [156] performed a GWAS for salinity avoidance/tolerance at the flowering stage and found that >73% of the identified loci overlapped with the existing salinity QTLs.

## 6. Genetic Engineering Tools for Salinity Tolerant Rice Plant Development

Due to its wide range of multigenicity, it is challenging to create comprehensive information regarding how plants react to stress, particularly salinity stress, at different developmental stages. Plant breeders using conventional approaches have achieved a certain level in producing saline tolerant crops. But the key factor of the meager results with conventional breeding was that the significance of genetic diversity in the wide range of genetic heritage is less [157]. Despite that, genetic engineering has improved enormously over the past two decades and has provided an essential base for understanding the improvement of many crops, including rice.

**Figure 4:**
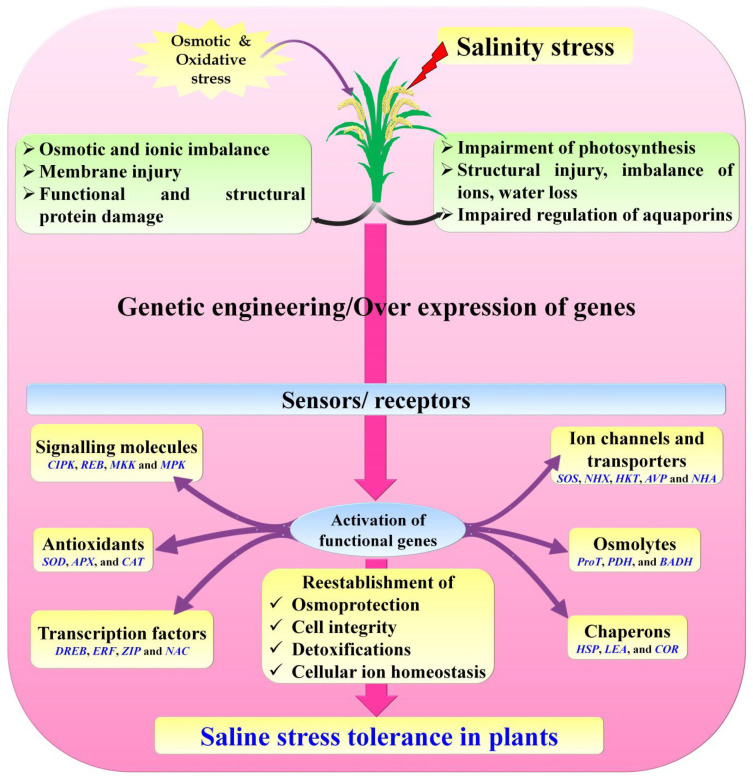
Alleviation of salt stress resistance via genetic engineering/overexpression in rice.

Overexpression of the genes is crucial and responsible for the salt stress tolerance in plants to enhance various proteins. For example, abscisic acid (ABA) ameliorated different proteins, including dehydrin, which alleviates saline stress and increases plant stress tolerance/avoidance [158]. In rice, overexpression of the genes viz., *OsARP*, *OsHAK5*, *OsKAT1*, *OsNHX1*, and *OsVP1* increased salt tolerance through enhanced growth of seedlings, root biomass production, strengthened the photosynthetic activity and ion homeostasis [159]. Plants such as Arabidopsis, rice, mustard, and tobacco are not capable of producing organic solutes under stress conditions. Overexpression of organic solutes through genetic engineering resulted in producing salt tolerance in crops viz., wheat [160], rice [161], tomato [162], potato [163], Arabidopsis [164,165], tobacco [166,167]. Ijaz et al. (2017) [168] reported that the overexpression of the *AnnSp2* gene in tomatoes exhibits high saline tolerance by enhancing the ABA ratio, which drastically reduced water loss and increased the frequency of closing stomata. Various genes are overexpressed through genetic engineering, which activates the functional genes in different crops, as is described in Figure 4. In Arabidopsis, various genes from different plant species were overexpressed and showed high saline tolerance, including the genes viz., *MtMYBS1* [169], *DREB2* [170,171], and *RD29A* [172]. About 38 classes of *DREB* genes transformed and overexpressed showed multi-stress, including saline resistance in various crop plants [173].

The Saline stress mechanism in plants is restrained by several genes that advance its tolerance [174]. Salinity tolerance improvement in plants involves various changes in different metabolic, physiochemical, and cellular machineries driven by gene-specific expressions. Candidates responsible for salinity stress or other TFs have numerous functions. It enhances the proline synthesis in plants and induces stomata closure to reduce the transpiration frequency, followed by producing a few essential stress-related defensive enzymes to increase saline stress tolerance. Several saline stress-related genes and TFs have been recognized, well categorized, genetically transformed in plants, and overexpressed against salinity stress and other abiotic stresses [174,175]. Significant progress has been made in producing saline stress-tolerant crops by incorporating genes and overexpression in plants through genetic transformation.

Genetic engineering of crops has changed, modified, or improved the genomes at the gene level accomplished through gene overexpression, RNA interference, or gene editing through the CRISPR/Cas9 system. The transgenic approach is essential technology for developing saline tolerant crops to withstand stress and harmful environmental circumstances [176,177,178]. Genetic transformation of identified salinity tolerant candidate genes in plants emphasizes their essential role in salt tolerance and high productivity. It is essential to improve saline-tolerant crops, and germplasm should be conserved as a heritage for upcoming generations. After the genetic transformation, tolerant and sensitive lines can be found and analyzed using transcriptome studies for differential gene expressions in leaves and roots or other tissues of the plants. The pattern of gene expression might include both up- and down-regulation of gene expression through various other experimental factors [179]. For example, in rice, Kurotani et al. [180] reported the salinity resistance was described to be linked with the elevated expression level of CYP94C-2b. Primarily, discovering the overall configurations of gene expression among saline susceptible and resistant progenies will be done through selecting highly expressed transcripts in plant tissues under stress, and were identified from major effects and contrast groups chosen from their pairwise interactions [179,181,182]. Moreover, at present, a wide range of genomic data has been developed and made publicly accessible for many plant species, especially species with the whole genome sequence already defined. Metagenomics analysis of various accessible genome sequences can increase the uniformity and quality of the reports [182].

In the past decade of the 21st century, the CRISPR/Cas9 genome editing method has been widely used for target-based plant genome editing [183]. However, the application of CRISPR/Cas9 genome editing has still not been implemented for many important crops. Recently, the *OsRR22* gene-targeted CRISPR/Cas9 genome editing method was reported in *O. sativa*, resulting in the significant enhancement of salinity tolerance [183]. More details about the CRISPR/Cas9 genome editing method were not described in this review. Analyzing the micro-RNA-associated genes has been a conventionally tough task owing to the unavailability of accurate target-based knockout tools. But it was succeeded by CRISPR/Cas9 genome editing method in various crops, including Arabidopsis [184], rice [185,186], and bentgrass [187]. Plant genome editing by sequence-specific nucleases is a potent genome editing method in which DNA is inserted or deleted, or relocated to the target site of the endogenous loci. This kind of promising approach will also be useful for the improvement of saline-tolerant crops.

## 7. Modern Multi-Omics Era to Improve the Salinity Tolerance

Recent advancements in biological sciences, particularly emerging sequencing and throughput omics approaches, have enabled the way to generate a huge spectrum of omics data, including transcriptome, epigenome, proteome, metabolome, hormonome, signalome, ionome, and phenome at the genome level from *O. sativa* grown under diverse climatic conditions [18,188,189,190]. Nevertheless, the public availability of a wide range of cell and molecular data researchers is still far away through categorizing and characterizing the molecular physiological responses of *O. sativa* datasets are extremely underutilized. Based on this point, the multi-omics approaches enable a significant way to incorporate each molecule of biological knowledge available into repositories which helps unravel the organisms at the cellular-molecular level, which could address the unparalleled way for a modern green revolution in stress biological research in several plant species.

In the first decade of the 21st century, genomic investigations in rice plants evolved gene reconnaissance and imparted detailed information about the unique and stress-related candidate players and their functional regulations. Furthermore, the scientific advancements and omics approaches allowed researchers to unveil the large-scale biological systems that delineated the different functionalities in plants, particularly cereals, in the current decade. These approaches are the main tool for developing omics resources in model plant species and also accelerated the extensive research at the translational level by integrating the data across food crops, mainly on rice [188,191]. On the other hand, the emerging advances in sequencing technologies have highly promoted the field of biological science, attributing the incomparable inventions in sequencing technologies and exploration of novel applications after complete genome sequencing. Notably, to elevate the genome sequencing projects, NGS approaches have given applications such as RNA-Seq for transcriptome and non-coding RNAome, Chromatin immunoprecipitation (ChIP)-seq analysis for analyzing the protein and DNA interaction, and WGS for genetic variation analysis and epigenomic dynamics by quantitative detection [192]. Overall, these advancements focus on signal transduction, transcriptional reprogramming, cell signaling, and transcriptional networks. In addition, several other new technologies have also emerged, in association with molecular signalome/interactome analyses used for unraveling the interactions formed by protein-nucleic acids (DNA and RNA) and protein-protein interactions (PPI) [193]. Plant hormonal profiling was used to understand cellular signaling [18,194], metabolic pathway analysis by metabolomics [195,196,197], and plant morpho-physiological changes were analyzed by phenomic approach [198]. These omics approaches and genomic datasets treat all the molecular mechanisms as interconnected elements of plant cellular and molecular physiological systems. Besides, bioinformatics plays a significant role in multi-omics associated research by directing a large number of experimental datasets at the whole-genome level, which efficiently obtained fruitful information. Together with obtained omics, results will provide knowledge and revise our perception and data exchange with other cereals [189,190,199,200]. Thus, the omics possession for multi-omics upshots from innovative analytical repositories consists of the decisive platform for systems analyses. The improvisation in research work has been done in the field of plant stress and molecular biology. Integrated omics and computational biology also improved the existing information. In recent years, integrated omics and computational biology have expanded the research horizon. Several new avenues for identifying abiotic stress-associated players, including salinity stress, and delineating their molecular-physiological mechanisms have also been developed.

### 7.1. Multi-Omics as a Significant Analysis to Address the Alleviation of Saline Stress

For dissecting the molecular machinery of abiotic stress, particularly salinity stress in rice plants, the available WGSs, understanding of the omics and bioinformatics tools, and research are the prerequisites to attain more molecular insights related to signaling and physiological pathways, complex defense signal from salinity stress, and cross-talks. Moreover, advancements in omics and computational biology-related rice research and resources have allowed us to delineate the molecular interactions and utilities of biological and cellular systems. For example, understand the rice metabolic pathways by KEGG (Kyoto Encyclopedia of Genes and Genomes), and this platform is generated by using integrated high-throughput omics (metabolomics), computational biology, and experimental datasets [201]. These omics and bioinformatic sources have been used to improve the researchers’ knowledge in understanding and implying to identify the abiotic stress, including salinity stress-responsive molecular biological systems insights that accelerate the gene discovery with their annotated information. Furthermore, these approaches help unravel the impact and outcome of the key candidate genes, proteins, and metabolites and their physiology, metabolic pathway regulations, gene network mapping, signaling molecules, and cross-talks responses to salinity stress and other abiotic stresses [16,34,202,203,204]. Up to our knowledge, these advancements are useful for evaluating the reverse genetics (from metabolites to proteins to genes) in salinity-treated rice plants.

Salinity stress often leads to causes osmotic stress [205]. To investigate the molecular mechanisms and metabolic pathways with their regulatory mechanisms in diverse responses to developmental changes, cellular differentiation, and abiotic stresses, including salinity stress which have been studied through ever-increasing integrated multi-omics approaches. Based on the integrated omics and plants systems analyses and the use of public repositories like PubMed search, gene families associated with the stress responses and cellular function with their molecular cross-talks were deciphered [18]. Compared to multi-omics approaches, phenomics data is crucial for identifying the genes, genome-wide association, and association mapping. Advancements in genomics and the appropriate availability of phenomics data limit the growth of genomics-assisted crop improvements [18,23]. The high throughput phenomics data extends the opportunity to catch the complex phenotypic variations used for tradition-based phenotypic selection. Further, the phenomics data, along with genomics, transcriptomic, and other omics approaches, will provide cost-effective, comprehensive trait data and promote the molecular dissection of traits associated with salt tolerance [23]. Overall, understanding the complexity of environmental plant responses to diverse abiotic stresses, mainly salinity stress, is the paramount task of the plant researchers, and additionally, facing the salinity-resistant rice plants is highly complicated. Hence, these multi-omics approaches coupled with computational biology unveil the salinity stress and other abiotic stress complexities and provide novel avenues.

### 7.2. Bioinformatic Databases

Multiple machines used in omics technologies generate a good quality of datasets, which were analyzed further, visualized, curated, and stored. Therefore, these in silico approaches are strongly coupled with bioinformatics tools, platforms, packages, online repositories, mathematical modeling, and programming languages that will aid the analyses, integration, and open the floodgates to researchers. Various bioinformatics tools have been used by the research community for analyzing a wide spectrum of multi-omics data in a more efficient, accurate, and reproducible method [206,207,208,209,210]. With the advancement of new bioinformatics tools and databases (Supplementary Table S3), researchers can identify and annotate the diverse abiotic stress-responsive genes responding significantly to the environmental changes, including saline stress [211], and also provide the eagle sight information. Specific bioinformatics knowledge could subsequently be harnessed to improve the crop species with enhanced production and exhibits abiotic and biotic stress tolerance. Developing the multi-omics strategies relies on in-depth analyses of different agricultural crop species during abiotic stress conditions. Extensive efforts have been made in all the cereal varieties with a tolerance for soil salinity and iron toxicities [211]. An omics tool is essential to unravel the novel avenues of an organism at the whole-genome level. After achieving genome sequencing, the aim is to identify and annotate the functionally important key players within the genome with the help of bioinformatics possessions [212]. Based on the obtained information, bioinformatics platforms are pivotal in analyzing deeper molecular biological insights.

## 8. Arbuscular Mycorrhizal Fungi (AMF) Are an Agricultural Factor in Alleviating the Saline Stress

**Figure 5:**
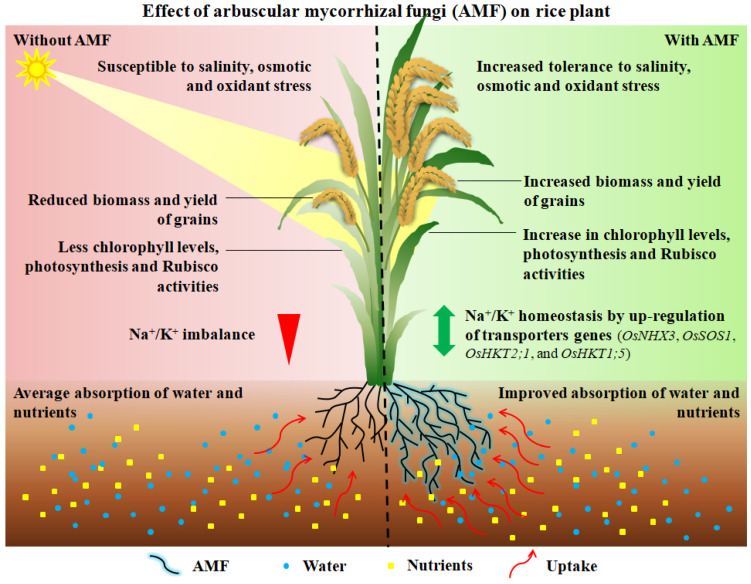
Effect of AMF on rice under saline stress condition. Differential responses with AMF and without AMF rice plants under saline stress. Na^+^ and K^+^ imbalance affects the plant’s physiological traits. AMF improves the water and essential nutrients uptake by roots, up-regulates the transporters, and also maintains the Na^+^ and K^+^ homeostasis. Salinity stress negatively affects photosynthesis, Rubisco activities, biomass, and yield. AMF positively regulates photosynthesis, Rubisco activities, biomass, and yield under salt stress conditions. Overall, AMF enhances the performance of the rice plant during saline stress.

There are plenty of beneficial endophytic fungi in the crop rhizosphere, while soil AMF was shown to improve plant development and productivity of rice in saline conditions [22,213,214,215,216,217]. AMF can establish symbionts with roots of various plants, including rice, and the developed mycorrhizal extraradical hyphae directly uptake the water and mineral nutrients from the soil to the host plant, thus enhancing the host’s ability to tolerate abiotic stress, mainly salt stress, through various physiological and molecular mechanisms [22,213,218,219,220]. In rice fields with high saline concentrations, eight arbuscular mycorrhizal fungal families were observed, and AMF diversity in rice roots was negatively correlated with soil salinity levels [215]. Under salt stress, AMF inoculation has been shown to increase chlorophyll levels and shoot K^+^/Na^+^ ratio, along with higher root biomass and grain yield of rice, dependent on specific AMF species or in a combination of two AMF species [216]. Fructose and proline accumulation for cell osmotic adjustment was more in mycorrhizal rice plants than in non-mycorrhizal rice plants under salt stress, thus maintaining the structure and functioning of chlorophyll [22]. In addition, mycorrhizal-containing rice plants had higher photosynthetic and RuBisCo activities than non-mycorrhizal plants exposed to salinity, thus improving the biomass production of rice [213]. The change in K^+^/Na^+^ ratio by mycorrhization was due to the up-regulates of the transporters genes (e.g., *OsNHX3*, *OsSOS1*, *OsHKT2;1*, and *OsHKT1;5*) expression involved in Na^+^/K^+^ homeostasis [214]. It is proposed that AMF is conducive to the extrusion of Na^+^ from the cytoplasm, that it fixes the vacuole, and enhances its recirculation from leaves to roots [214,221]. AMF thus ameliorates the salinity stress, and the positive effects are described in Figure 5. This mechanism is a guarantee for vigorous growth of rice under soli saline conditions. Therefore, the application and importance of AMF to rice in the field is an alternative and efficient approach to alleviating saline damage and maintaining the yield of rice grown in saline soil.

## 9. Conclusions and Future Perspectives

In abiotic stresses, salinity stress is one of the predominant stressors which dauntingly affect the overall plant growth and yield of rice plants across the world. In addition, saline stress is a highly complex process whose elements are still far from clear because several enzymes and metabolites have overlapping cellular compartments owing to the possible divergence at tolerance levels. Therefore, to enhance rice yield during salinity stress, a new tool and method are needed to understand the molecular mechanisms, develop new cultivars, enhance yield and stress tolerance/adaptations, and solve this roadblock. This comprehensive review summarizes the key findings and investigations in advancing genetic engineering, biotechnological tools, breeding, agricultural practicing factors, high-throughput omics, and computational biology in rice systems under salinity stress conditions. Further, these integrated omics and bioinformatic approaches have enabled us to unravel the many avenues of salinity stress associated with players with their mechanisms and functional regulations. In addition, we also discussed the candidate genes, TFs, plant growth hormones, ABA-dependent and ABA-independent networks, osmoprotectants, signaling molecules, specialized databases, and the role of AMF in regulating salinity stress responses in rice plants.

Genomics-based breeding is fruitful knowledge for improving salt tolerance in rice. Though, new breeding strategies, such as MAGIC populations and GS, also need to be used simultaneously to optimize the understanding of genetic mechanisms and discovery of QTLs/genes for complex traits like salinity tolerance. Genetic engineering in terms of overexpression of candidate players and biotechnological tools, mainly on genome/gene editing through CRISPR/Cas9 technology, has a crucial role in improving salt-tolerant rice with enhanced yield production and nutrition.

In furtherance of these advancements, specific concentration should also be paid to the interactions between salinity and other abiotic stress dynamisms. This will unravel further avenues regarding the cross-talks between salinity stress-responsive players and the reported abiotic stress signaling pathways. On the other hand, our understanding of these advancements in salinity stress needs to be further refined. In addition, available literature, stress-responsive gene families, TFs, cellular and molecular functions, signaling networks, and integrated omics datasets used to develop bioinformatic interfaces/platforms are used to unveil the effective salinity tolerance mechanisms and also derive useful strategies for crop improvements. Overall, this review will also provide a future perspective by pointing out the remaining questions regarding the role of discussed research findings in salinity stress tolerance. It will enable us to refine and significantly enhance the yield (quality and quantity) of rice for the benefit of humanity.

## Data Availability

Not applicable.

## Supplementary Materials

The following supporting information can be downloaded at: https://www.mdpi.com/article/10.3390/biology11071022/s1, Table S1: Salinity stress-responsive genes and their function; Table S2: Salinity stress-responsive TFs and their functions; Table S3: Rice bioinformatics databases and tools for multiple omics analyses.
