# Supplementary material for: Multi-Omics and Integrative Approach towards Understanding Salinity Tolerance in Rice: A Review
Source: Biology (Basel). 2022 Jul 7;11(7):1022. doi: 10.3390/biology11071022 (PMC9312129; doi:10.3390/biology11071022)
Supplement: Supplementary file 1 [file biology-11-01022-s001.zip › biology-1786644-supplementary.pdf]

Supplementary Table S1. Rice bioinformatic databases and tools for multiple omics analyses

Tools & Databases	Database description	Reference
RiceSRTFDB	Rice TFs	[1]
STIFDB2	Plant stress responsive genes	[2]
PSPDB	Plant stress related proteins	[3]
QlicRice	Rice abiotic stress responsive QTLs	[4]
RiceNetDB	Rice genome annotated information	[5]
RiceXPro	Gene expression profiles of tissues, organs and hormones	[6]
Stress2TF	Curated stress responsive TF informations	[7]
Plant ontology	Resource of plant anatomy, morphology, growth, and development to plant genomic data	[8]
Phytozome v10.2	Comparative genomic analysis	[9]
PlantPReS	Plant proteome response to stress	[10]
PtRFdb	Plant tRNA-derived fragments	[11]
GRASSIUS	TFs information	[12]
O. sativa genome DB –PlantGDB	EST, cDNA, PUT, and proteins.	[13]
BGI rice Database	Transcriptome analysis, small RNA, sequence alignment, *de novo* genome assembly, functional annotation, evolutionary analysis.	[14]
Rice SNP seek Database	Information for rice genotype, phenotype and variety.	[15]
R-Oryzabase	DNA sequences, literature, phenotype description and classification of rice.	[16]
Integrated Genome Browser	Genomics analytic platform	[17]
Gramene	Comparative functional genomics	
Plant-Reactome Gramene Pathways	Analysis of plant pathways	[18]
Gramene QTL Database	QTL prediction	[19]
Expression Atlas	Annotated gene information	[20]
RAP-DB	Rice genome structure and function	[21]
KEGG	Pathway analysis	[22]
STRING	Protein- protein interaction and gene protein interaction	[23]
MCDRP	Curated rice proteins	[24]
Phospho Rice	Rice specific phosphorylation sites	[25]
funRiceGene	Comprehensive database of functionally characterized rice genes	[26]
PlantCARE and	cis-acting regulatory elements prediction	[27,28]
PLACE		
ExPASy - Protparam	Protein properties prediction	[29]
RiceFOX DB	Hormanome profile	[30]
PPDB	Plant proteomics	[31]
RIPP-DB	Plant phosphoproteome database	[32]
Oryza PG-DB	Rice proteogenomics database	[33]
GOEAST	Gene ontology annotation	[34]
Circos	Comparative genome visualization	[35]
Cytoscape web	Network visualization tool-modelled	[36]
PRIDE inspector	Visualize mass spectrometry (MS) proteomics data	[37]

Supplementary Table S2: Salinity stress responsive genes and its function

Gene	Gene description	Inducible to	Result	Reference
Rab 16A	LEA group of proteins	Drought, Salinity, Cold	Rice plants response to salinity tolerance	[38,39]
GDH	Glutamate dehydrogenase	Salinity, Drought, Cold	Abiotic stress tolerance	[40]
OsNAC2	TF	Salinity, Cold. Drought	Abiotic stress tolerance	[41]
OsMYB6	TF	Drought, Salinity	Salinity tolerance in rice	[42]
P5CS	Pyrroline carboxylate synthetase (proline synthesis)	Salinity	Accumulation of Proline in young cladodes	[43]
Mn-SOD	Superoxide dismutase	Salinity	Salinity tolerance	[44]
GS2	Glutamine synthetase (chloroplast)	Salinity, Cold	Salinity stress and chilling tolerance	[45]
OsCDPK7	TF (calcium-dependent protein kinase)	Salinity, Cold, Drought	Salinity, cold and drought stress tolerance	[46]
OsCDPK			Positive regulation of the stress signaling pathways, increase in salinity, cold and drought tolerance	
Mt1D	Manitol-1-phosphate dehydrogenase (manital synthesis)	Salinity	Salinity tolerance in rice	[47]
codA	Choline synthase (Betaine biosynthesis)	Salinity, Drought	Enhanced enhanced biomass production	[48]
OsNAC5	TF	Salinity, Cold, Drought	Detoxification, redox homeostasis, and macromolecule fortification	[49]
SNAC1	TF (Stress responsive NAC)	Salinity	Salinity tolerance	
DREB1B/CBF1	TF	Salinity, Drought	Salinity and drought tolerance	[50]
DREB1A/CBF3		Salinity	Salinity tolerance	[41]
ZFP179		Salinity	Salinity tolerance	[51]
OsCDPK21	TF (calcium-dependent protein kinase)	Salinity	Positive regulation of stress signaling pathways, increase in salinity tolerance and ABA	[52]
MAPK5	Mitogen-activated protein kinase	Salinity, Cold and Drought	Salinity, drought and cold stress tolerance	[53]
MAPK44		Salinity	Salinity tolerance	[54]
SAPK4	Serine-threonine protein kinase	Salinity	Salinity tolerance in rice	[55]
OsNHX1	Sodium/hydrogen exchanger	Salinity	Salinity tolerance, maintained growth at 200 mM NaCl and increased biomass production	[56,57]
OsSIKI	Stress-induced protein kinase gene 1 (Receptor-like kinases)	Drought, Salinity	Salinity and drought stress tolerance	[58]
OsSIK2	Stress-induced protein kinase gene 2 (Receptor-like kinases)	Drought, Salinity	Abiotic stress tolerance and early leaf development and senescence phenotype	[59]
SOS2	Salinity Overly Sensitive 2	Drought, Salinity	Exchange of Na^+/H^+ in plasma membrane of yeast (*Saccharomyces cerevisiae*) cells and reduced their	[60]

			Na^+ content	
SDIR1	Salinity- and drought-induced ring finger 1	Drought and Salinity	Decrease water loss likely due to their enhanced stomata response	[61]
DSR2	DUF966 stress-repressive gene 2	Drought, Cold, Salinity, Heat, Oxidative	Salinity and drought stress tolerance and decreased ABA sensitivity	[62]
OsNCED3	9-cisEpoxycarotenoid dioxygenase (rice stress-inducible promoter)	Drought, Salinity	ABA biosynthesis, roots and leaves enhancement under drought and salinity conditions	[63]
OsCYP2	Cyclophilin 2	Salinity	Controls ROS level, involved in PEG, heat, cold, or ABA signal pathways	[64]
OsPYL/RCA R5	Pyrabactin Resistance 1 Like/Regulatory Components of ABA receptors	Drought, Salinity	Abiotic stress tolerance in rice, inhibits plant growth, and modulates gene expression	[65]
OsLEA3-2	Late embryogenesis abundant proteins	Drought, Salinity	Elevated growth performance under salinity/osmotic stress conditions	[66]
OsOAT	Ornithine ***δ***-amino-transferase)	Drought, Salinity, Oxidative	Involved in proline (Pro) and arginine (Arg) metabolism, increased δ-OAT activity, glutathione (GSH) and ROS content	[67]
OsSAP	Stress associated Protein	Multiple abiotic stresses including salinity	Induces the ubiquitination, ROS scavenging activities, redox sensing and hormone metabolism	[68,69]

Supplementary Table S3: Salinity stress responsive TFs and their functions

TF Family	Genes	Function	Abiotic tolerance	Reference
bZIP	bZIP23, bZIP71	Increase sensitivity to exogenous ABA at both germination and post-germination stages, improving the stress resistance of crops	Drought, salinity	[70,71]
	bZIP4	Regulates the ABA synthesis development	Salinity	[72]
	bZIP110	Regulating absorption of Na^+ and proline accumulation	Salinity	[73]
	ABF2	Function in osmotic stress signaling	Salinity	[74]
	ABF3		Salinity	
	ABF4	Participate in multiple stress responses	Drought, Salinity, Cold	
	bZIP44,62,78	Differentially regulate expressions of ABA and stress- responsive genes	Drought, Salinity, Cold	[75]
	bZIP132	Reduce ABA sensitivity, increased water loss	Salinity, Drought	[76]
	ABI5	Regulation of adaptive stress response	Salinity	[77]
NAC	NAC29	Improve physiological traits, reduced H_2O_2 accumulation	salinity, drought	[78]
	NAC022	Na^+ accumulation js less in roots and shoots, increases proline and soluble sugar contents	Salinity, drought, and ABA	[79]
	NAC2	Lateral root development and stress tolerance	Salinity	[80]
	NAC019,055,072	Upregulation of stress inducible	Drought, Salinity	[81]
	NAC4	Regulation of plant stresses	Drought, salinity, Cold	[82]
	NAC6	Root system development and yield, regulates the stress tolerance	Drought, salinity	[83-85]
	NAC066	Increases proline and soluble sugar contents	Salinity, H_2O_2	[86]
	NAC2	Stress tolerance	Salinity, Drought	[87]
	NAC4,11,13,24,29,30, 37,47,51, 52,59,69,76,80,86,87,112	Playe a role in abiotic stress tolerance, signal transduction pathway regulation	Salinity, Drought, metal, cold, temperature, UV, flooding	[88]
	NAC032	Promotes senescence and accumulation of H_2O_2, stress tolerance	Salinity, mannitol	[89]
bHLH	bHLH035	Mediates seed germination and seedling recovery	Salinity	[90]
	bHLH22	Increase osmotic potential and accumulation of secondary metabolites	Salinity, Drought	[91]
	bHLH26,87,94,105, 110,116,127,129	Multiple abiotic stress tolerance and transduction signal pathway regulation	Salinity, Drought, metal, cold, temperature, UV, flooding	[88]
	bHLH38	Regulate osmotic balance, stress-induced oxidation damage	Salinity, Drought	[92]
	MYC2	Regulate proline biosynthesis	Salinity	[93]
	MYB6	Elevated CAT and SOD activities,	Salinity, Drought	[94]

MYB		salt stress tolerance		
	MYB91	Rice tolerance to abiotic stress	Salinity	[95]
	MYB15	Enhanced ABA sensitivity, reduced water loss rate, increased ABA biosynthesis	Drought, Salinity	[96]
	MYB76	Stress tolerance and plant physiology processes	Salinity	[97]
	MYB92		Salinity, Cold	
	MYB117		Drought, Salinity	
	MYB76	Response to ionic stresses, improve stress resistance	Salinity	[98]
	MYB3R-2	Increased tolerance to Salt	Freezing, Drought, and Salinity	[99]
	MYB48-1	Increase Salinity tolerance	Salinity, Drought	[100]

	MYB118	Regulate osmotic stress	Salinity, Drought	[101]
CBF/DREB	DREB2	Salinity and dehydration stress in roots and stems, transcript accumulation and alternative splicing regulation	Drought, Salinity, Cold	[102]
	DREB1F, DREB2A, DREB5A & DREB6B	Increased the number of transcripts in roots and stem	Salinity, Cold, Dehydration and ABA	[103]
	DREB1A	Produce transgenic monocots that are tolerant abiotic stress	Cold, Salinity	[104]
	DREB1C		Drought, Salinity, Cold	
	DREB2C	Negative impact of several ABA/stress-responsive genes, involve physical interaction	Cold, Salinity	[105]
	DREB1F	Regulation of ABA-dependent pathway, Stress tolerance	Drought, Salinity,	[106]
AP2/ERF	ERF3	Enhanced resistance to high salinity and dehydration stresses	Salinity, Drought	[107]
	EREBP1,9,10,11,36,39,49,75, 87,104,132,145,147	Multiple abiotic stress tolerance mechanisms and signal transduction pathway regulation	Salinity, Drought, metal, cold, temperature, UV, flooding	[88]
WRKY	WRKY30	Higher antioxidant activities and lower ROS contents, increases soluble sugar and proline contents	Salinity	[108]
	WRKY93	Increase stress tolerance, maintain membrane stability and played a major role superior agricultural trait of SR3 through promoting root development	Salinity	[109]
	WRKY13,54	Abiotic stress response and root development	Drought, Salinity	[110]
	WRKY83	Alterations in physiological parameters such as proline, malondialdehyde (MDA) and electrolyte leakage after stress treatments, increase proline accumulation	Salinity	[111]
	WRKY4,20,25,31,53,68,76, 79,90,103,104	Multiple abiotic stress tolerance mechanisms and signal transduction pathway regulation	Salinity, Drought, metal, cold, temperature, UV, flooding	[88]
	WRKY33	Improved Salinity stress tolerance than the wild type under abiotic stress condition.	Salinity	[112]

	SAP1, 2, 5, 6, 7, 8, 9, 11,	Developmental regulation, signal transduction,	Salinity, Drought, metal, cold, temperature, UV,	[69]

ZF-HD	17	translation, biosynthesis of amino acids, energy metabolism	flooding	
	Zat7	Salinity stress tolerance to transgenic plants, defense response	Salinity	[113]
	CaZF	Potential plant salt-tolerance and osmotolerance determinant	Salinity	[114]
	ZHD10,12,14	Multiple abiotic stress tolerance mechanisms and signal transduction pathway regulation, plant differentiation	Salinity, Drought, metal, cold, temperature, UV, flooding	[88]
HSF	HSF1-25	Transcriptional, post-transcriptional, translational and post-translational activators of abiotic stressors, regulate hormonal signal transduction	Salinity, Drought, metal, cold, heat, UV, flooding	[115]
Others	HARDY	Improves water use efficiency, increase in leaf biomass and bundle sheath cells, stress tolerance	Drought, Salinity	[116]

References

1. Priya, P. and Jain, M., 2013a. RiceSRTFDB: a database of rice transcription factors containing comprehensive expression, cis-regulatory element and mutant information to facilitate gene function analysis. Database, 2013.
2. Naika, M., Shameer, K., Mathew, O.K., Gowda, R. and Sowdhamini, R., 2013. STIFDB2: an updated version of plant stress-responsive transcription factor database with additional stress signals, stress-responsive transcription factor binding sites and stress-responsive genes in Arabidopsis and rice. Plant and Cell Physiology, 54(2), pp.e8-e8.
3. Kumar, S.A., Kumari, P.H., Sundararajan, V.S., Suravajhala, P., Kanagasabai, R. and Kishor, P.K., 2014. PSPDB: plant stress protein database. Plant molecular biology reporter, 32(4), pp.940-942.
4. Smita, S., Lenka, S.K., Katiyar, A., Jaiswal, P., Preece, J. and Bansal, K.C., 2011. QlicRice: a web interface for abiotic stress responsive QTL and loci interaction channels in rice. Database, 2011.
5. Liu, L. et al. An integrative bioinformatics framework for genome-scale multiple level network reconstruction of rice. Journal of integrative bioinformatics. 10, 94-102 (2013).
6. Sato, Y. et al. RiceXPro Version 3.0: expanding the informatics resource for rice transcriptome. Nucleic Acids Res. 41, D1206–D1213 (2013b).
7. Zhang, X., Yao, C., Fu, S., Xuan, H., Wen, S., Liu, C., Li, F., Liu, A., Bi, S., Zhang, S. and Li, S., 2018. Stress2TF: a manually curated database of TF regulation in plant response to stress. Gene, 638, pp.36-40.
8. Cooper, L. and Jaiswal, P., 2016. The plant ontology: a tool for plant genomics. In Plant Bioinformatics (pp. 89-114). Humana Press, New York, NY.
9. Goodstein, D. M. et al. Phytozome: a comparative platform for green plant genomics. Nucleic Acid Res. 40, D1178–D1186 (2012).
10. Mousavi, S.A., Pouya, F.M., Ghaffari, M.R., Mirzaei, M., Ghaffari, A., Alikhani, M., Ghareyazie, M., Komatsu, S., Haynes, P.A. and Salekdeh, G.H., 2016. PlantPReS: A database for plant proteome response to stress. Journal of proteomics, 143, pp.69-72.
11. Gupta, N., Singh, A., Zahra, S. and Kumar, S., 2018. PtRFdb: a database for plant transfer RNA-derived fragments. Database, 2018.
12. Yilmaz, A. et al. GRASSIUS: a platform for comparative regulatory genomics across the grasses. Plant Physiol. 149, 171–180 (2009).
13. Duvick, J. et al. PlantGDB: a resource for comparative plant genomics. Nucleic Acids Res. 36, D959–D965 (2008).
14. Zhao, W. et al. BGI-RIS: an integrated information resource and comparative analysis workbench for rice genomics. Nucleic Acids Res. 32, D377–D382 (2003).
15. Alexandrov, N., Tai, S., Wang, W., Mansueto, L., Palis, K., Fuentes, R.R., Ulat, V.J., Chebotarov, D., Zhang, G., Li, Z. and Mauleon, R., 2014. SNP-Seek database of SNPs derived from 3000 rice genomes. Nucleic acids research, 43(D1), pp.D1023-D1027.
16. Kurata, N. & Yamazaki, Y. Oryzabase. An Integrated Biological and Genome Information Database for Rice. Plant Physiol. 140 (2006).
17. Freese, N.H., Norris, D.C. and Loraine, A.E., 2016. Integrated genome browser: visual analytics platform for genomics. Bioinformatics, 32(14), pp.2089-2095.
18. Tello-Ruiz, M. K. et al. Gramene 2016: comparative plant genomics and pathway resources. Nucleic Acids Res. 44, 1133–1140 (2016).
19. Ni, J. et al. Gramene QTL database: development, content and applications. Database, Article ID bap005 (2009).
20. Petryszak, R. et al. Expression Atlas update - an integrated database of gene and proteinexpression in humans, animals and plants. Nucleic Acids Res. 44, 746–752 (2016).
21. Sakai, H. et al. Rice Annotation Project Database (RAP-DB): An Integrative and Interactive Database for Rice Genomics. Plant Cell Physiol. 54, 1–11 (2012).
22. Kanehisa M, Goto S. KEGG: Kyoto Encyclopedia of Genes and Genomes. Nucleic Acids Res. 2000;28(1):27–30. PMID: 10592173.
23. Szklarczyk, D. et al. The STRING database in 2017: quality-controlled protein–protein association networks, made broadly accessible. Nucleic Acids Res. 45, D362–D368 (2017).
24. Raghuvanshi, S., Gour, P. & Joseph, S.V. Manually Curated Database of Rice Proteins (MCDRP), a database of digitized experimental data on rice. Current Plant Biol. 8, 26–30 (2016).
25. Que, S. et al. PhosphoRice: a meta-predictor of rice specific phosphorylation sites. Plant Methods. 8, 5(2012).
26. Yao, W., Li, G., Yu, Y. & Ouyang, Y. funRiceGenes dataset for comprehensive understanding and application of rice functional genes. Giga Sci. 7, gix119 (2017).
27. Higo, K., Ugawa, Y., Iwamoto, M. & Korenaga, T. Plant cis-acting regulatory DNA elements (PLACE) database: 1999. Nucleic Acid Res. 27, 297-300 (1999).
28. Lescot, M. et al. PlantCARE, a database of plant cis-acting regulatory elements and a portal to tools for in silico analysis of promoter sequences. Nucleic Acid Res. 30, 325-327 (2002).
29. Gasteiger, E. et al. Protein Identification and Analysis Tools on the ExPASy Server. In Walker, J. M. (ed), The Proteomics Protocols Handbook, Springer, NewYork, pp. 571– 607 (2005).

30. Sakurai, T. et al. RiceFOX: a database of Arabidopsis mutant lines overexpressing rice full-length cDNA that contains a wide range of trait information to facilitate analysis of gene function. Plant and cell physiol. 52, 265-273 (2010).
31. Sun, Q. et al. PPDB, the plant proteomics database at Cornell. Nucliec. Acid. Res. 37, D969-D974 (2008).
32. Nakagami, H. et al. Large scale comparative phosphoproteomics identifies conserved phosphorylation sites in plants. Plant Physiol. 153, 1161-1174 (2010).
33. Helmy, M., Tomita, M. & Ishihama, Y. OryzaPG-DB: rice proteome database based on shotgun proteogenomics. BMC Plant Biol. 11, 63 (2011).
34. Zheng, Q. & Wang, X. J. GOEAST: a web-based software toolkit for Gene Ontology enrichment analysis. Nucleic Acids Res. 36, W358-W363 (2008).
35. Krzywinski, M., Schein, J., Birol, I., Connors, J., Gascoyne, R., Horsman, D., Jones, S.J. and Marra, M.A., 2009. Circos: an information aesthetic for comparative genomics. Genome research, 19(9), pp.1639-1645.
36. Lopes, C.T., Franz, M., Kazi, F., Donaldson, S.L., Morris, Q. and Bader, G.D., 2010. Cytoscape Web: an interactive web-based network browser. Bioinformatics, 26(18), pp.2347-2348.
37. Wang, R., Fabregat, A., Ríos, D., Ovelleiro, D., Foster, J.M., Côté, R.G., Griss, J., Csordas, A., Perez-Riverol, Y., Reisinger, F. and Hermjakob, H., 2012. PRIDE Inspector: a tool to visualize and validate MS proteomics data. Nature biotechnology, 30(2), pp.135- 137.
38. Ganguly, M., Datta, K., Roychoudhury, A., Gayen, D., Sengupta, D.N. and Datta, S.K., 2012. Overexpression of Rab16A gene in indica rice variety for generating enhanced Salinity tolerance. Plant signaling & behavior, 7(4), pp.502-509.
39. Basu, S. and Roychoudhury, A., 2014. Expression profiling of abiotic stress-inducible genes in response to multiple stresses in rice (Oryza sativa L.) varieties with contrasting level of stress tolerance. BioMed research international, 2014.
40. Skopelitis, D.S., Paranychianakis, N.V., Paschalidis, K.A., Pliakonis, E.D., Delis, I.D., Yakoumakis, D.I., Kouvarakis, A., Papadakis, A.K., Stephanou, E.G. and Roubelakis- Angelakis, K.A., 2006. Abiotic stress generates ROS that signal expression of anionic glutamate dehydrogenases to form glutamate for proline synthesis in tobacco and grapevine. The Plant Cell, 18(10), pp.2767-2781.
41. Hu, H., Dai, M., Yao, J., Xiao, B., Li, X., Zhang, Q. and Xiong, L., 2006. Overexpressing a NAM, ATAF, and CUC (NAC) transcription factor enhances drought resistance and Salinity tolerance in rice. Proceedings of the National Academy of Sciences, 103(35), pp.12987-12992.
42. Liu, C., Chen, K., Zhao, X., Wang, X., Shen, C., Zhu, Y., Dai, M., Qiu, X., Yang, R., Xing, D. and Pang, Y., 2019. Identification of genes for salt tolerance and yield-related traits in rice plants grown hydroponically and under saline field conditions by genome- wide association study. Rice, 12(1), p.88.
43. Silva-Ortega, C.O., Ochoa-Alfaro, A.E., Reyes-Agüero, J.A., Aguado-Santacruz, G.A. and Jiménez-Bremont, J.F., 2008. Salinity stress increases the expression of p5cs gene and induces proline accumulation in cactus pear. Plant Physiology and Biochemistry, 46(1), pp.82-92.
44. Eyidoğan, F.I., Öktem, H.A. and Yücel, M., 2003. Superoxide dismutase activity in Salinity stressed wheat seedlings. Acta physiologiae plantarum, 25(3), pp.263-269.
45. Hoshida, H., Tanaka, Y., Hibino, T., Hayashi, Y., Tanaka, A., Takabe, T. and Takabe, T., 2000. Enhanced tolerance to Salinity stress in transgenic rice that overexpresses chloroplast glutamine synthetase. Plant molecular biology, 43(1), pp.103-111.
46. Saijo, Y., Hata, S., Kyozuka, J., Shimamoto, K. and Izui, K., 2000. Over- expression of a single Ca2+- dependent protein kinase confers both cold and Salinity/drought tolerance on rice plants. The Plant Journal, 23(3), pp.319-327.
47. Li, Z.C., X.C. Zhang, L. Zhang, B.C. Thuang, C.L. hang, G.Y. Wang and Y.C. Fu, 2004. Expression of MtD1 gene in transgenic rice leads to enhanced Salinity tolerance. J. china Agric. Univ, 9: 38-43
48. Li, H., Wang, Z., Ke, Q., Ji, C.Y., Jeong, J.C., Lee, H.S., Lim, Y.P., Xu, B., Deng, X.P. and Kwak, S.S., 2014. Overexpression of codA gene confers enhanced tolerance to abiotic stresses in alfalfa. Plant Physiology and Biochemistry, 85, pp.31-40.
49. Hu, H., You, J., Fang, Y., Zhu, X., Qi, Z. and Xiong, L., 2008. Characterization of transcription factor gene SNAC2 conferring cold and Salinity tolerance in rice. Plant molecular biology, 67(1-2), pp.169-181.
50. Oh, S.J., Song, S.I., Kim, Y.S., Jang, H.J., Kim, S.Y., Kim, M., Kim, Y.K., Nahm, B.H. and Kim, J.K., 2005. Arabidopsis CBF3/DREB1A and ABF3 in transgenic rice increased tolerance to abiotic stress without stunting growth. Plant physiology, 138(1), pp.341-351.
51. Sun, S.J., Guo, S.Q., Yang, X., Bao, Y.M., Tang, H.J., Sun, H., Huang, J. and Zhang, H.S., 2010. Functional analysis of a novel Cys2/His2-type zinc finger protein involved in Salinity tolerance in rice. Journal of experimental botany, 61(10), pp.2807-2818.
52. Asano, T., Hakata, M., Nakamura, H., Aoki, N., Komatsu, S., Ichikawa, H., Hirochika, H. and Ohsugi, R., 2011. Functional characterisation of OsCPK21, a calcium-dependent protein kinase that confers Salinity tolerance in rice. Plant molecular biology, 75(1-2), pp.179-191.
53. Xiong, L. and Yang, Y., 2003. Disease resistance and abiotic stress tolerance in rice are inversely modulated by an abscisic acid–inducible mitogen-activated protein kinase. The Plant Cell, 15(3), pp.745-759.
54. Jeong, M.J., Lee, S.K., Kim, B.G., Kwon, T.R., Cho, W.S., Park, Y.T., Lee, J.O., Kwon, H.B., Byun, M.O. and Park, S.C., 2006. A rice (Oryza sativa L.) MAP kinase gene, OsMAPK44, is involved in response to abiotic stresses. Plant Cell, Tissue and Organ Culture, 85(2), pp.151-160.
55. Diédhiou, C.J., Popova, O.V., Dietz, K.J. and Golldack, D., 2008. The SNF1-type serine- threonine protein kinase SAPK4 regulates stress-responsive gene expression in rice. BMC plant biology, 8(1), pp.1-13.

56. Fukuda, A., Nakamura, A., Tagiri, A., Tanaka, H., Miyao, A., Hirochika, H. and Tanaka, Y., 2004. Function, intracellular localization and the importance in Salinity tolerance of a vacuolar Na+/H+ antiporter from rice. Plant and cell physiology, 45(2), pp.146-159.
57. Chen, M., Chen, Q., Niu, X., Zhang, R., Lin, H., Xu, C., Wang, X., Wang, G. and Chen, J., 2007. Expression of OsNHX1 gene in maize confers Salinity tolerance and promotes plant growth in the field. Plant Soil and Environment, 53(11), p.490.
58. Ouyang, S. Q., Liu, Y. F., Liu, P., Lei, G., He, S. J., Ma, B., et al. (2010). Receptor-like kinase OsSIK1 improves drought and Salinity stress tolerance in rice (Oryza sativa) plants. Plant J. 62: 316–329.
59. Chen, L. J., Wuriyanghan, H., Zhang, Y. Q., Duan, K. X., Chen, H. W., Li, Q. T., et al. (2013). An S-domain receptor-like kinase, OsSIK2, confers abiotic stress tolerance and delays dark-induced leaf senescence in rice. Plant Physiol. 163: 1752–1765.
60. Martinez-Atienza, J., Jiang, X., Garciadeblas, B., Mendoza, I., Zhu, J. K., Pardo, J. M. and Quintero, F. J. (2007). Conservation of the Salinity Overly Sensitive Pathway in Rice. Plant Physiol. 143(2): 1001-1012.
61. Gao, T., Wu, Y., Zhang, Y., Liu, L., Ning, Y., Wang, D., Tong, H., Chen, S., Chu, C., Xie, Q. (2011). OsSDIR1 overexpression greatly improves drought tolerance in transgenic rice. Plant Mol Biol 76: 145–156.
62. Luo, C., Guo, C., Wang, W., Wang, L., Chen, L. (2013). Overexpression of a new stress- repressive gene OsDSR2 encoding a protein with a DUF966 domain increases Salinity and simulated drought stress sensitivities and reduces ABA sensitivity in rice. Plant Cell Rep 33: 323–336.
63. Bang, S. W., Park, S. H., Jeong, J. S., Kim, Y. S., Jung, H., Ha, S. H., Kim, J. K. (2013). Characterization of the stress-inducible OsNCED3 promoter in different transgenic rice organs and over three homozygous generations. Planta 237(1):211-24.
64. Ruan, S. L., Ma, H. S., Wang, S. H., Fu, Y. P., Xin, Y., Liu, W. Z., Wang, F., Tong, J. X., Wang, S. Z., Chen, H. Z. (2011). Proteomic identification of OsCYP2, a rice cyclophilin that confers Salinity tolerance in rice (Oryza sativa L.) seedlings when overexpressed. BMC Plant Biology. 11.
65. Kim, H., Lee, K., Hwang, H., Bhatnagar, N., Kim, D.-Y., Yoon, I. S., et al. (2014). Over expression of PYL5 in rice enhances drought tolerance, inhibits growth, and modulates gene expression. J. Exp. Bot. 65: 453–464.
66. Duan, J., and Cai, W. (2012). OsLEA3-2, an abiotic stress induced gene of rice plays a key role in Salinity and drought tolerance. PLoS ONE 7: e45117.
67. You, J., Hu, H., and Xiong, L. (2012). An ornithine δ-aminotransferase gene OsOAT confers drought and oxidative stress tolerance in rice. Plant Sci 197: 59–69.
68. Krishnan, S. R., Muthuramalingam, P., Mohan, C., & Ramesh, M. (2018). Emerging Trends Of A20/An1 Zinc-Finger Proteins In Improving Rice Productivity Under Abiotic Stress. Rice Science: Biotechnological and Molecular Advancements, 1.
69. Muthuramalingam, P., Jeyasri, R., Selvaraj, A., Kalaiyarasi, D., Aruni, W., Pandian, S.T. K., & Ramesh, M. (2020b). Global transcriptome analysis of novel stress associated protein (SAP) genes expression dynamism of combined abiotic stresses in Oryza sativa (L.). Journal of Biomolecular Structure and Dynamics, 1-12.
70. Xiang, Y., Tang, N., Du, H., Ye, H. and Xiong, L., 2008. Characterization of OsbZIP23 as a key player of the basic leucine zipper transcription factor family for conferring abscisic acid sensitivity and salinity and drought tolerance in rice. Plant physiology, 148(4), pp.1938-1952.
71. Liu, C., Mao, B., Ou, S., Wang, W., Liu, L., Wu, Y., Chu, C. and Wang, X., 2014. OsbZIP71, a bZIP transcription factor, confers salinity and drought tolerance in rice. Plant molecular biology, 84(1-2), pp.19-36.
72. Ma, H., Liu, C., Li, Z., Ran, Q., Xie, G., Wang, B., Fang, S., Chu, J. and Zhang, J., 2018. ZmbZIP4 contributes to stress resistance in maize by regulating ABA synthesis and root development. Plant physiology, 178(2), pp.753-770.
73. Xu, Z., Ali, Z., Xu, L., He, X., Huang, Y., Yi, J., Shao, H., Ma, H. and Zhang, D., 2016. The nuclear protein GmbZIP110 has transcription activation activity and plays important roles in the response to salinity stress in soybean. Scientific reports, 6, p.20366.
74. Choi, H.I., Hong, J.H., Ha, J.O., Kang, J.Y. and Kim, S.Y., 2000. ABFs, a family of ABA- responsive element binding factors. Journal of Biological Chemistry, 275(3), pp.1723-1730.
75. Liao, Y., Zou, H.F., Wei, W., Hao, Y.J., Tian, A.G., Huang, J., Liu, Y.F., Zhang, J.S. and Chen, S.Y., 2008a. Soybean GmbZIP44, GmbZIP62 and GmbZIP78 genes function as negative regulator of ABA signaling and confer salt and freezing tolerance in transgenic Arabidopsis. Planta, 228(2), pp.225-240.
76. Liao, Y., Zhang, J.S., Chen, S.Y. and Zhang, W.K., 2008b. Role of soybean GmbZIP132 under abscisic acid and salt stresses. Journal of Integrative Plant Biology, 50(2), pp.221-230
77. Zou, M., Guan, Y., Ren, H., Zhang, F. and Chen, F., 2008. A bZIP transcription factor, OsABI5, is involved in rice fertility and stress tolerance. Plant molecular biology, 66(6), pp.675-683.
78. Xu, Z., Wang, C., Xue, F., Zhang, H. and Ji, W., 2015. Wheat NAC transcription factor TaNAC29 is involved in response to salt stress. Plant Physiology and Biochemistry, 96, pp.356-363.
79. Hong, Y., Zhang, H., Huang, L., Li, D. and Song, F., 2016. Overexpression of a stress- responsive NAC transcription factor gene ONAC022 improves drought and salt tolerance in rice. Frontiers in Plant Science, 7, p.4.
80. He, X.J., Mu, R.L., Cao, W.H., Zhang, Z.G., Zhang, J.S. and Chen, S.Y., 2005. AtNAC2, a transcription factor downstream of ethylene and auxin signaling pathways, is involved in salt stress response and lateral root development. The Plant Journal, 44(6), pp.903-916.

81. Tran, L.S.P., Nakashima, K., Sakuma, Y., Simpson, S.D., Fujita, Y., Maruyama, K., Fujita, M., Seki, M., Shinozaki, K. and Yamaguchi-Shinozaki, K., 2004. Isolation and functional analysis of Arabidopsis stress-inducible NAC transcription factors that bind to a drought-responsive cis- element in the early responsive to dehydration stress 1 promoter. The Plant Cell, 16(9), pp.2481-2498.
82. Meng, C., Cai, C., Zhang, T. and Guo, W., 2009. Characterization of six novel NAC genes and their responses to abiotic stresses in Gossypium hirsutum L. Plant Science, 176(3), pp.352-359.
83. Nakashima, K., Tran, L.S.P., Van Nguyen, D., Fujita, M., Maruyama, K., Todaka, D., Ito, Y., Hayashi, N., Shinozaki, K. and Yamaguchi- Shinozaki, K., 2007. Functional analysis of a NAC- type transcription factor OsNAC6 involved in abiotic and biotic stress- responsive gene expression in rice. The Plant Journal, 51(4), pp.617-630.
84. Lee, D.K., Chung, P.J., Jeong, J.S., Jang, G., Bang, S.W., Jung, H., Kim, Y.S., Ha, S.H., Choi, Y.D. and Kim, J.K., 2017. The rice Os NAC 6 transcription factor orchestrates multiple molecular mechanisms involving root structural adaptions and nicotianamine biosynthesis for drought tolerance. Plant Biotechnology Journal, 15(6), pp.754-764.
85. Chung, P.J., Jung, H., Do Choi, Y. and Kim, J.K., 2018. Genome-wide analyses of direct target genes of four rice NAC-domain transcription factors involved in drought tolerance. BMC genomics, 19(1), p.40.
86. Yuan, X., Wang, H., Cai, J., Bi, Y., Li, D. and Song, F., 2019. Rice NAC transcription factor ONAC066 functions as a positive regulator of drought and oxidative stress response. BMC plant biology, 19(1), p.278.
87. Shen, J., Lv, B., Luo, L., He, J., Mao, C., Xi, D. and Ming, F., 2017. The NAC-type transcription factor OsNAC2 regulates ABA-dependent genes and abiotic stress tolerance in rice. Scientific reports, 7, p.40641.
88. Muthuramalingam, P., Krishnan, S. R., Saravanan, K., Mareeswaran, N., Kumar, R., & Ramesh, M. (2018a). Genome-wide identification of major transcription factor superfamilies in rice identifies key candidates involved in abiotic stress dynamism. *Journal of Plant Biochemistry and Biotechnology*, *27*(3), 300-317.
89. Mahmood, K., El-Kereamy, A., Kim, S.H., Nambara, E. and Rothstein, S.J., 2016. ANAC032 positively regulates age-dependent and stress-induced senescence in Arabidopsis thaliana. Plant and Cell Physiology, 57(10), pp.2029-2046.
90. Chen, H.C., Cheng, W.H., Hong, C.Y., Chang, Y.S. and Chang, M.C., 2018. The transcription factor OsbHLH035 mediates seed germination and enables seedling recovery from Salinity stress through ABA-dependent and ABA-independent pathways, respectively. Rice, 11(1), p.50.
91. Waseem, M. and Li, Z., 2019. Dissecting the Role of a Basic Helix-Loop-Helix Transcription Factor, SlbHLH22, Under Salinity and Drought Stresses in Transgenic Solanum lycopersicum L. Frontiers in plant science, 10, p.734.
92. Qiu, J.R., Huang, Z., Xiang, X.Y., Xu, W.X., Wang, J.T., Chen, J., Song, L., Xiao, Y., Li, X., Ma, J. and Cai, S.Z., 2020. MfbHLH38, a Myrothamnus flabellifolia bHLH transcription factor, confers tolerance to drought and salinity stresses in Arabidopsis. BMC Plant Biology, 20(1), pp.1-14.
93. Verma, D., Jalmi, S.K., Bhagat, P.K., Verma, N. and Sinha, A.K., 2019. A bHLH transcription factor, MYC2, imparts Salinity intolerance by regulating proline biosynthesis in Arabidopsis. The FEBS Journal.
94. Tang, Y., Bao, X., Zhi, Y., Wu, Q., Guo, Y., Yin, X., Zeng, L., Li, J., Zhang, J., He, W. and Liu, W., 2019. Overexpression of a MYB family gene, OsMYB6, increases drought and salinity stress tolerance in transgenic rice. Frontiers in plant science, 10, p.168.
95. Zhu, N., Cheng, S., Liu, X., Du, H., Dai, M., Zhou, D.X., Yang, W. and Zhao, Y., 2015. The R2R3-type MYB gene OsMYB91 has a function in coordinating plant growth and Salinity stress tolerance in rice. Plant Science, 236, pp.146-156.
96. Ding, Z., Li, S., An, X., Liu, X., Qin, H. and Wang, D., 2009. Transgenic expression of MYB15 confers enhanced sensitivity to abscisic acid and improved drought tolerance in Arabidopsis thaliana. Journal of Genetics and Genomics, 36(1), pp.17-29.
97. Liao, Y., Zou, H.F., Wang, H.W., Zhang, W.K., Ma, B., Zhang, J.S. and Chen, S.Y., 2008c. Soybean GmMYB76, GmMYB92, and GmMYB177 genes confer stress tolerance in transgenic Arabidopsis plants. Cell research, 18(10), pp.1047-1060.
98. He, Y., Li, W., Lv, J., Jia, Y., Wang, M. and Xia, G., 2012. Ectopic expression of a wheat MYB transcription factor gene, TaMYB73, improves salinity stress tolerance in Arabidopsis thaliana. Journal of Experimental Botany, 63(3), pp.1511-1522.
99. Dai, X., Xu, Y., Ma, Q., Xu, W., Wang, T., Xue, Y. and Chong, K., 2007. Overexpression of an R1R2R3 MYB gene, OsMYB3R-2, increases tolerance to freezing, drought, and salt stress in transgenic Arabidopsis. Plant physiology, 143(4), pp.1739-1751.
100. Xiong, H., Li, J., Liu, P., Duan, J., Zhao, Y., Guo, X., Li, Y., Zhang, H., Ali, J. and Li, Z., 2014. Overexpression of OsMYB48-1, a novel MYB-related transcription factor, enhances drought and salinity tolerance in rice. PloS one, 9(3), p.e92913.
101. Du, Y.T., Zhao, M.J., Wang, C.T., Gao, Y., Wang, Y.X., Liu, Y.W., Chen, M., Chen, J., Zhou, Y.B., Xu, Z.S. and Ma, Y.Z., 2018. Identification and characterization of GmMYB118 responses to drought and Salinity stress. BMC plant biology, 18(1), pp.1-18
102. Egawa, C., Kobayashi, F., Ishibashi, M., Nakamura, T., Nakamura, C. and Takumi, S., 2006. Differential regulation of transcript accumulation and alternative splicing of a DREB2 homolog under abiotic stress conditions in common wheat. Genes & genetic systems, 81(2), pp.77-91.
103. Konzen, E.R., Recchia, G.H., Cassieri, F., Caldas, D.G.G., Berny Mier y Teran, J.C., Gepts, P. and Tsai, S.M., 2019. DREB genes from common bean (Phaseolus vulgaris L.) show broad to specific abiotic stress responses and distinct levels of nucleotide diversity. International journal of genomics, 2019.

104. Dubouzet, J.G., Sakuma, Y., Ito, Y., Kasuga, M., Dubouzet, E.G., Miura, S., Seki, M., Shinozaki, K. and Yamaguchi-Shinozaki, K., 2003. OsDREB genes in rice, Oryza sativa L., encode transcription activators that function in drought- , high- salt- and cold- responsive gene expression. The Plant Journal, 33(4), pp.751-763.
105. Lee, S.J., Kang, J.Y., Park, H.J., Kim, M.D., Bae, M.S., Choi, H.I. and Kim, S.Y., 2010. DREB2C interacts with ABF2, a bZIP protein regulating abscisic acid-responsive gene expression, and its overexpression affects abscisic acid sensitivity. Plant physiology, 153(2), pp.716-727.
106. Wang, Q., Guan, Y., Wu, Y., Chen, H., Chen, F. and Chu, C., 2008. Overexpression of a rice OsDREB1F gene increases salt, drought, and low temperature tolerance in both Arabidopsis and rice. Plant molecular biology, 67(6), pp.589-602.
107. Zhang, G., Chen, M., Li, L., Xu, Z., Chen, X., Guo, J. and Ma, Y., 2009. Overexpression of the soybean GmERF3 gene, an AP2/ERF type transcription factor for increased tolerances to Salinity, drought, and diseases in transgenic tobacco. Journal of experimental botany, 60(13), pp.3781-3796.
108. Zhu, D., Hou, L., Xiao, P., Guo, Y., Deyholos, M.K. and Liu, X., 2019. VvWRKY30, a grape WRKY transcription factor, plays a positive regulatory role under salinity stress. Plant Science, 280, pp.132-142.
109. Qin, Y., Tian, Y. and Liu, X., 2015. A wheat salinity-induced WRKY transcription factor TaWRKY93 confers multiple abiotic stress tolerance in Arabidopsis thaliana. Biochemical and biophysical research communications, 464(2), pp.428-433.
110. Zhou, Q.Y., Tian, A.G., Zou, H.F., Xie, Z.M., Lei, G., Huang, J., Wang, C.M., Wang, H.W., Zhang, J.S. and Chen, S.Y., 2008. Soybean WRKY- type transcription factor genes, GmWRKY13, GmWRKY21, and GmWRKY54, confer differential tolerance to abiotic stresses in transgenic Arabidopsis plants. Plant biotechnology journal, 6(5), pp.486-503.
111. Wu, M., Liu, H., Han, G., Cai, R., Pan, F. and Xiang, Y., 2017. A moso bamboo WRKY gene PeWRKY83 confers salinity tolerance in transgenic Arabidopsis plants. Scientific reports, 7(1), pp.1-16.
112. Li, H., Gao, Y., Xu, H., Dai, Y., Deng, D. and Chen, J., 2013. ZmWRKY33, a WRKY maize transcription factor conferring enhanced Salinity stress tolerances in Arabidopsis. Plant Growth Regulation, 70(3), pp.207-216.
113. Ciftci-Yilmaz, S., Morsy, M.R., Song, L., Coutu, A., Krizek, B.A., Lewis, M.W., Warren, D., Cushman, J., Connolly, E.L. and Mittler, R., 2007. The EAR-motif of the Cys2/His2-type zinc finger protein Zat7 plays a key role in the defense response of Arabidopsis to salinity stress. Journal of Biological Chemistry, 282(12), pp.9260-9268.
114. Jain, D., Roy, N. and Chattopadhyay, D., 2009. CaZF, a plant transcription factor functions through and parallel to HOG and calcineurin pathways in Saccharomyces cerevisiae to provide osmotolerance. PloS one, 4(4), p.e5154.
115. Muthuramalingam, P., Jeyasri, R., Bharathi, R. K. A. S., Suba, V., Pandian, S. T. K., & Ramesh, M. (2020a). Global integrated omics expression analyses of abiotic stress signaling HSF transcription factor genes in Oryza sativa L.: An in silico approach. *Genomics*, *112*(1), 908-918.
116. Karaba, A., Dixit, S., Greco, R., Aharoni, A., Trijatmiko, K.R., Marsch-Martinez, N., Krishnan, A., Nataraja, K.N., Udayakumar, M. and Pereira, A., 2007. Improvement of water use efficiency in rice by expression of HARDY, an Arabidopsis drought and salt tolerance gene. Proceedings of the National Academy of Sciences, 104(39), pp.15270-15275.
